# Variation in the Molecular Phenotype of *βglu‐1*, an Insect Defence‐Related Beta‐Glucosidase Gene, in Two Transcontinental *Picea* Species

**DOI:** 10.1111/mec.70120

**Published:** 2025-10-23

**Authors:** Ernest T. Y. Wu, Tin Hang Hung, Nadir Erbilgin, Aziz Ullah, Āris Jansons, Hisham A. A. Ali, Sonya M. Clegg, John J. MacKay

**Affiliations:** ^1^ Department of Biology University of Oxford Oxford UK; ^2^ Department of Renewable Resources University of Alberta Edmonton Alberta Canada; ^3^ Latvian State Forest Research Institute “Silava” Salaspils Latvia

**Keywords:** *Choristoneura fumiferana*, evolutionary response, gene expression, insect defence, *Picea abies*, *Picea glauca*

## Abstract

Forest trees face threats from many insect pest species, underscoring the importance of understanding their defence mechanisms for survival. In a North American conifer species, *Picea glauca* , gene expression of *β* *glu‐1* produces phenolic compounds (acetophenones) that defend against its insect defoliator, *Choristoneura fumiferana* . *β* *glu‐1* is also expressed in a Eurasian conifer species, *Picea abies* , although no major insect defoliators are present within the species' natural range. We compared the range‐wide variation of *β* *glu‐1* transcript levels from foliage samples of *P. glauca* in North America and *P. abies* in Europe using RT‐qPCR and targeted transcriptome sequencing. *β* *glu‐1* transcript levels were highly correlated between the two methods, with large transcript level variation being detected within and between populations in both species and one of the *β* *glu‐1* gene forms being significantly differentially expressed in *P. glauca* . Importantly, *β* *glu‐1* transcript levels in *P. glauca* varied longitudinally and were positively associated with mean annual precipitation and *C. fumiferana* outbreak class, which has historically higher outbreak frequency and severity in eastern compared to western populations. In *P. abies* , *β* *glu‐1* transcript levels were associated with annual temperature range. Overall, these results enhance our understanding of potential adaptive variation in acetophenone defences in *P. glauca* , while the factors influencing *β* *glu‐1* transcript variation in *P. abies* require further investigation.

## 1 Introduction

The ability of long‐lived tree species to develop adaptive defences against their natural enemies is essential to the longevity and ecosystem functioning of forests (Delvas et al. 2011; Wiggins et al. 2016). Trees can develop physical (anatomical and morphological) and chemical (e.g., phenolics, flavonoids, terpenes) defences to reduce attack from insect herbivory (Mithöfer and Boland 2012). The production of chemical defences may either be induced upon attack or occur constitutively, being synthesised and stored in certain tissues over time without costly activation mechanisms following herbivory (Lankau and Strauss 2008; Mithöfer and Maffei 2016; Van Zandt 2007). Acetophenones are a common chemical defence mechanism used in some conifer species, including *Picea glauca* (Moench) Voss, to protect themselves against major insect defoliators, such as *Choristoneura fumiferana* (Clemens), in North America (Mageroy et al. 2015; Méndez‐Espinoza et al. 2018; Parent et al. 2017). In its natural range, *C. fumiferana* defoliates many species of *Picea*, such as *P. glauca* and *Abies balsamea* . Variation in *P. glauca* resistance against *C. fumiferana* is linked to the constitutive (i.e., preformed) concentrations of acetophenones and transcript levels of the genes involved (Lamara et al. 2018). This defence trait varies among *P. glauca* populations of eastern Canada (Parent et al. 2017), but whether this variation extends across the entire species range or to other species of *Picea* is unknown.

Considerable phenotypic variation has been observed in defence traits within *P. glauca* (Hall et al. 2011; Parent et al. 2017; Parent et al. 2020; see also Whitehill et al. 2021). Population‐level variation in defence traits could reflect biogeographical history, quantitative and qualitative differences in the selective pressure from their pests, as well as other phenological and climate factors. A better understanding of the interpopulation variation in defences in *P. glauca* against *C. fumiferana* can inform how host species may adapt to emerging biotic threats, such as the expanding geographical ranges of insect pests and higher frequencies of outbreaks as a result of the changing climate (Bellemin‐Noël et al. 2021; Tai and Carroll 2022; Weed et al. 2013).

Here, we report on the transcriptional differences in acetophenone defences in two transcontinental boreal conifer species, *P. glauca* and *Picea abies* (L.) H. Karst. Both tree species have distinct contemporary population structures because of their biogeographical histories (Gaston 2003). Range shifts, disjunctions and coalescence over the last 20,000 years of the Pleistocene glaciation have resulted in eastern and western *P. glauca* haplotypes in North America (de Lafontaine et al. 2010). *P. abies* has a disjunct north and south distribution in Europe (Tollefsrud et al. 2008), with three distinct genetic clusters (Alpine, Nordic and Carpathians; Chen et al. 2019) that diverged 15 million years ago and went through a bottleneck during the last ice age. These distinct populations may have significant genetic and phenotypic differences in their functional traits, including insect defence traits.

In *P. glauca* , two acetophenones, piceol and pungenol, have been verified to decrease larval survival and pupal mass and delay the development of *C. fumiferana* (Parent et al. 2020). These acetophenones work in combination with monoterpenes to resist *C. fumiferana* , with the acetophenones impeding growth and reproduction, and the monoterpenes causing mortality (Ullah et al. 2024). *P. glauca* and *C. fumiferana* likely persisted in a glacial refugia during the Last Glacial Maximum, with their current population structures forming during post‐glacial recolonisation (Lumley et al. 2020). The natural ranges of both species are only found in North America, with the geographic distribution of *C. fumiferana* subpopulations closely matching the phylogeographic pattern of white spruce. In *P. abies* , which has a Eurasian distribution, acetophenone concentration is suggested to be an indicator of plant stress to abiotic conditions (Lokke 1990), but no studies have verified the acetophenones to be effective against any of its natural enemies. *P. abies* may be using acetophenones for a different purpose, or the acetophenones may be a retained evolutionary trait.

The glycosylation and subsequent hydrolysation of secondary metabolites are common in plants; for example, they play an important role in regulating plant defence (Le Roy et al. 2016). The release of bioactive acetophenones from their glycosylated forms involves β‐glucosidase 1 (βGLU‐1) (Mageroy et al. 2015). The aglycone acetophenones are derived from the general phenolpropanoid pathway and are synthesised by unknown enzymes. A UDP‐sugar dependent glucosyltransferase, encoded by *Ugt5b*, then glycosylates the acetophenones (i.e., piceol to picein and pungenol to pungenin) for storage and bioaccumulation in the cell (Mageroy et al. 2017). βGLU‐1 then functions to hydrolyse picein and pungenin back to their bioactive counterparts. In *P. glauca* , variation in aglycone acetophenone levels and relative insect resistance correlates with the expression of *βglu‐1* (Parent et al. 2018; Mageroy et al. 2017). Several complete and partial *βglu‐1* forms have recently been detected in *P. glauca* and *P. abies* with different expression levels; notably, there are five and three highly expressed full‐length gene forms in *P. glauca* and *P. abies* respectively (Hung et al. 2024).

Phenotypic variation in aglycone acetophenone levels in *P. glauca* has been shown to have additive genetic architecture (Lamara et al. 2018) and is highly heritable (Méndez‐Espinoza et al. 2018). Acetophenone concentrations in *Picea* foliage are contingent upon βGLU‐1 activity and an adequate supply of picein and pungenin. The glycosylated acetophenones may accumulate to high levels (e.g., picein levels can be five times higher than piceol in *P. glauca* ), suggesting that they are not limiting for the release of acetophenones in most individuals (Mageroy et al. 2015). Therefore, it is likely that *βglu‐1* expression drives variation in acetophenone concentrations and can be used as a proxy for defence phenotype. A study on eastern North American *P. glauca* populations observed that *βglu‐1* transcript levels have distinct spatial patterns (Parent et al. 2017). Comparing the gene expression of this trait across the entire species range may reveal how the phenotype responds to differing biotic or abiotic pressures across the landscape (Sork 2018; Lasky et al. 2014). Gene expression studies in other species such as *Arabidopsis* (Kuśnierczyk et al. 2007), maize (Kollner et al. 2008) and *Nicotiana* (Wu et al. 2008) have shown a direct relationship between resistance to herbivores and transcriptional variation in defensive genes involved in the biosynthesis of glucosinolates, terpenes and other secondary metabolites (Brachi et al. 2015; Broekgaarden et al. 2011).

To date, there has been no range‐wide study of *βglu‐1* expression or acetophenone concentrations in *P. glauca* or *P. abies* . Therefore, to understand the biogeography of the acetophenone defence, we aim to (1) develop methods for *βglu‐1* transcript level quantification based on recently characterised gene models by Hung et al. (2024); (2) determine *βglu‐1* transcript levels across the natural species ranges of *P. glauca* and *P. abies* ; (3) assess whether transcript levels of *βglu‐1* show geographical patterns that correlate with abiotic or biotic variables.

## 2 Materials and Methods

### 2.1 Plant Materials and RNA Isolation

**FIGURE 1:**
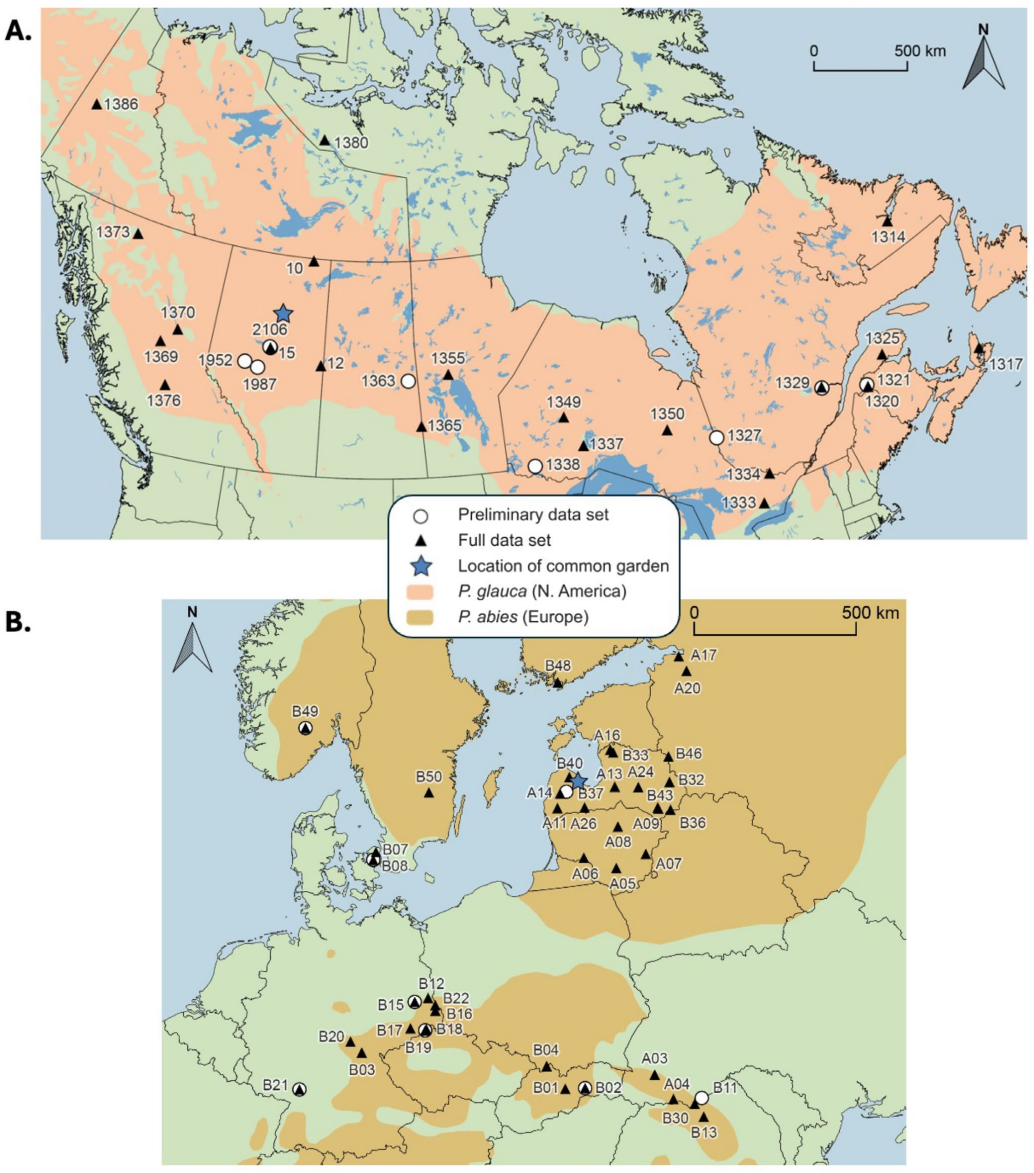
Map showing ranges and sampled provenances of (A) *Picea glauca* and (B) *Picea abies* in both the preliminary (2021; circle markers) and full (2022; triangular markers) sample set.

Foliage samples were collected from common gardens located at Calling Lake, Alberta, Canada (N 55° 17′, W 133° 09′) for *P. glauca* , planted in 1982, and Saldus, Latvia (N 56° 50′, E 22° 29′) for *P. abies* , planted in the 1970s. A preliminary sample set was obtained in 2021 for method optimisations and targeted transcriptome sequencing, with 39 individuals from nine populations of *P. glauca* (Figure 1A) and 38 individuals from eight populations of *P. abies* (Figure 1B). Major sampling was conducted from the same common gardens between July 11 and 24, 2022, to obtain the full sample set, with 436 individuals from 22 populations of *P. glauca* (Figure 1A) and 302 individuals from 43 populations of *P. abies* (Figure 1B; see Table S1 for details). Samples were collected in July as the acetophenone production starts in the spring and remains elevated until July (Mageroy et al. 2017), but decreases later in the season. Each foliage sample consisted of 3–5 shoot tips of current‐year growth from the top half of the tree crown. The samples were collected and immediately wrapped in aluminium foil and frozen on dry ice (−76°C) for later transfer to deep freezers (−80°C).

The needles were removed from the twigs using liquid nitrogen and ground in 2 mL Eppendorf tubes with two steel balls using a Milli Mixer 440 (Retsch, Haan, NRW, Germany). Total RNA was isolated using a NEB Monarch Total RNA Miniprep Kit (New England BioLabs, https://international.neb.com/products/t2010‐monarch‐total‐rna‐miniprep‐kit) following the manufacturer's protocol with modifications to the sample input volume to maximise yield. The RNA was eluted in 50 μL of nuclease‐free water and stored at −80°C; the concentration was determined by using a NanoDrop 1000 (Thermo Scientific, http://www.thermoscientific.com/) and Qubit 4 Fluorometer with an RNA BR kit (Thermo Scientific). An RNA IQ kit for Qubit was used to assess RNA quality for a third of the samples. RNA quality scores were calculated by taking the ratio of large or structured RNA to small, fragmented RNA. All samples with yields > 4000 ng were used in subsequent downstream analysis. First‐strand cDNA was synthesised from 500 ng of RNA for samples in the preliminary sample set and 200 ng of RNA for samples from the full sample set using a Maxima First Strand cDNA Synthesis Kit for RT‐qPCR (K1642, Thermo Fisher Scientific, United Kingdom).

### 2.2 *βglu‐1* Transcript Level Quantification Using qPCR Methods

Levels of mRNA transcripts were determined using reverse transcription‐quantitative PCR (RT‐qPCR). For the target gene, *βglu‐1* (Genbank accession # KJ780719 for *P. glauca* and MG049736 for *P. abies* ), the three primer pairs used to amplify different regions of the gene were described in Mageroy et al. (2015). The primer pair for the reference gene, *Ef1a* (Genbank accession # BT112014 for *P. glauca* and AJ132534 for *P. abies* ), was designed using Primer3Plus (http://www.bioinformatics.nl/cgi‐bin/primer3plus/primer3plus.cgi) software (see Table S2 for all primer sequences). All primer pairs were experimentally tested for both species using PCR with a HotStart PCR Master Mix (Thermo Scientific) and verified using gel electrophoresis that a single amplicon at the expected size (80 to 130 bp) was obtained. The PCR products were verified by Sanger sequencing with 97.6%, 97.6%, 96.0% and 95.9% matches to the expected *βglu‐1*‐E1, *βglu‐1*‐E2, *βglu‐1*‐E3 and *Ef1a* reference sequences, respectively, in *P. glauca* and 98%, 96%, 98% and 98% for matching the expected *βglu‐1*‐E1, *βglu‐1*‐E2, *βglu‐1*‐E3 and *Ef1a* reference sequences, respectively, for *P. abies* .

The RT‐qPCR mixtures consisted of a PowerUp SYBR Green MasterMix (Thermo Scientific; 5 μL), 500 nM (0.5 μL) each of forward and reverse primers, 10 ng (1 μL) of cDNA and nuclease‐free water for a 10 μL reaction. Amplifications were carried out in a StepOnePlus thermocycler (Applied Biosystems) for the Preliminary sample set and a 384‐well QuantStudio5 thermocycler (Applied Biosystems) for the full sample set, both following the standard cycling method for primers (Tm > 60°C).

Quantifications of the RT‐qPCR products were obtained from standard curves by use of serial dilutions (ranging from 10^8^ to 10^0^ copies) of synthetic gene fragments (Twist Biosciences; https://www.twistbiosciences.com; see Table S3 for sequence information). The threshold cycle, known as C‐half or C_1/2_ (adapted from Rutledge and Stewart 2010), was determined for each RT‐qPCR reaction by calculating the inflection point on the sigmoidal curve of the amplification profile. We first calculated the corrected fluorescence for each cycle by removing the background fluorescence (average of the first 10 cycles) from the raw fluorescence values. Since the inflection point is also the mid‐point of the amplification profile, the reaction would have reached its maximum efficiency at the C_1/2_ cycle. Therefore, we calculated C_1/2_ by taking (max corrected fluorescence value—min corrected fluorescence value)/2. A standard curve was then constructed for each primer pair, whereby the C_1/2_ value of each dilution was plotted onto a linear regression and the number of *βglu‐1* amplicons of each sample was determined by relating the C_1/2_ of the sample to the standard curve. The transcript level of *βglu‐1* was then divided by the sample to average ratio of the reference gene (*Ef1a*) transcript level for normalisation.

### 2.3 *β* *glu‐1* Transcript Level Quantification Using Targeted Transcriptome Sequencing

Transcriptome sequencing was done using *P. glauca* and *P. abies* samples from the preliminary sample set only. A further second‐strand cDNA was synthesised with a NEBNext Ultra II Non‐Directional RNA Second Strand Synthesis Module (E6111, New England Biolabs, United Kingdom). Using the NEBNext Ultra II Library Prep Kit for Illumina (#E7645, New England Biolabs), all double‐stranded cDNA was purified, end‐repaired, dA‐tailed and then ligated with adaptors for Illumina (#E6609, New England Biolabs). The sub‐libraries were then amplified as well as barcoded using the ProFlex PCR System (Thermo Fisher Scientific). The PCR reactions (25 μL) consisted of the following: 12.5 μL Kapa HiFi HotStart ReadyMix (Kapa Biosystems, United Kingdom), 2.5 μL NEBNext Multiplex Oligos for Illumina (10 μM) (#E6609, New England Biolabs), 10 μL adaptor‐ligated cDNA.

Using the Long Insert Protocol (manual version 5.02), we conducted the hybridisation capture with myBaits Custom 1–20 K Kit (Daciel Arbor Biosciences, Ann Arbour, MI, USA). The probe set was developed in a previous study (Picea_hung_p1.0) (Hung et al. 2024) to target *βglu‐1‐* and *UGT5b*‐like genes as well as predicted single‐copy genes in both species. Hung et al. 2024 produced two local assemblies Piabi_c.1.0 and Pigla_c1.0, respectively, with 1443 and 1552 gene models characterised using both ab initio prediction and transcript evidence from transcriptome assemblies PIAB_v1 and PIGL_v1. Among the gene models, 77 were found to be *βglu‐1*‐like and 36 *UGT5b*‐like genes in *P. glauca* and 62 *βglu‐1*‐like and 30 *UGT5b*‐like genes in *P. abies* . In addition, there were 48 high‐confidence single‐copy genes shared between both species. Therefore, these 161 and 140 genes in *P. glauca* and *P. abies* , respectively, were expected to be captured by the probes.

The target‐enriched sub‐library was then amplified using the same reaction mix as the barcoding amplification above. All sub‐libraries were pooled and normalised. All purification steps used 1.2× AMPure XP (Beckman Coulter, CA, USA). The thermal cycling profile was as follows: 94°C 3 min, 10 (barcoding) or 28 (post‐capture) × [94°C 30 s, 65°C 2 min 30 s], 65°C 5 min.

Nanopore libraries were then constructed by using the Ligation Sequencing Kit Chemistry 12 (SQK‐LSK112, Oxford Nanopore Technologies, United Kingdom) and the ~200 fmol pooled library. We sequenced the Nanopore libraries on R10.3 flow cells (FLO‐MIN111) on an in‐house GridION system (Oxford Nanopore Technologies, UK) at the Department of Biology, University of Oxford.

All Nanopore reads were base‐called and demultiplexed from their raw electrical signals by using Guppy v6.0.0, they were trimmed for Nanopore and Illumina adaptors, then split for chimeras using Porechop 0.2.4 (https://github.com/rrwick/Porechop/releases). Reads were aligned to the gene models of local genome assemblies (Piabi_c1.0 and Pigla_c1.0) (Hung et al. 2024), which were previously curated using this probe set, with splicing awareness (‐axe splice) in minimap 2.22 (Li 2018). Read quantification was then performed using Salmon v1.5.2 (Roberts and Pachter 2013) with the ‘‐ont’ flag to improve quantification based on the error profiles of Nanopore alignments. Salmon was chosen for its ‘quasi‐mapping’ method, which computes the likelihood of a read mapping to each transcript based on its sequence similarity, thus allowing for more accurate quantifications of expression levels when multi‐mapped reads are present because of sequence similarity or alternative splicing (Deschamps‐Francoeur et al. 2020). Out of the 161 and 140 targeted genes in *P. glauca* and *P. abies* , genes with zero read counts were removed, and reads were then normalised using DESeq2 (Love et al. 2014) in R version 4.3.2. A log likelihood ratio test was used to detect differentially expressed genes across the population.

### 2.4 Comparisons Between qPCR and Transcriptome Sequencing Results

**FIGURE 2:**
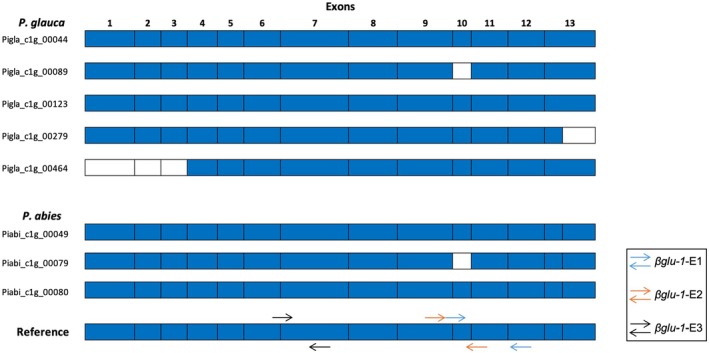
Exonic structure of all full‐length *βglu‐1* gene forms based on the local assemblies of *Picea glauca* and *Picea abies* as described in Hung et al. (2024) and mapped to the reference *βglu‐1* gene model (KJ780719.1; 1521 bp). Blue indicates the presence of the exon in the gene model; no colour indicates the exon is truncated or missing. Binding sites for each of the three primer pairs used in RT‐qPCR are also shown.

To choose RT‐qPCR primer pairs that amplify *βglu‐1* consistently across all provenances, a multiple measures ANOVA (MANOVA) was run on the preliminary sample set to determine primer pairs that produced significantly different transcript levels from each other. The transcript levels determined by the significantly different RT‐qPCR primer pairs were then correlated with the targeted transcriptome sequencing read counts of each of the full‐length and near full‐length gene forms, three for *P. abies* (Piabi_c1g_00049, 00079 and 00080; Figure 2) and five gene forms in *P. glauca* (Pigla_c1g_00464, 00279, 00123, 00089 and 00044; Figure 2) and the sum of all gene forms at the individual tree level for each species using Spearman's correlation.

### 2.5 Spatial Analysis of Geographical Patterns in *β* *glu‐1* Transcript Levels

To explore geographical patterns in RT‐qPCR results of the full sample set, we first tested the effect of latitude and longitude with provenance as the random variable, using a linear mixed effects model: lmer (gene expression ~ latitude + longitude + (1|Provenance)). We then built a dendrogram of relationships of *βglu‐1* transcript levels using hierarchical clustering in the hclust function (R package cluster; Langfelder and Horvath 2012). We colour‐coded the dendrogram branches based on population origin using the contMap function (R package phytools; Revell 2012). Next, we tested for spatial autocorrelation of *βglu‐1* transcript levels for the different populations using the Moran. I function (R package ape; Paradis and Schliep 2019). We also tested for pairwise dissimilarity between *βglu‐1* transcript levels in *P. glauca* populations using non‐numeric multidimensional scaling (NMDS) (function MetaMDS, Euclidian method, R package vegan; Dixon 2003) and permutation‐based multivariate analysis of variance (perMANOVA) (function adonis2, R package vegan). NMDS was not conducted in *P. abies* because of the small sample size of some provenances.

### 2.6 Biotic and abiotic drivers of gene expression

We gathered bioclimatic variables (Bioclim) from WorldClim version 2.1 climate data for 1970–2000 at the 30 s resolution (Fick and Hijmans 2017) for all *P. glauca* and *P. abies* provenances included in this study. We chose BIO1 (mean annual temperature), BIO7 (temperature annual range), BIO12 (mean annual precipitation) and BIO15 (precipitation seasonality) as the four representative variables that affect tree growth and verified that none of these variables exceeded a correlation threshold of 0.9 for all populations using the Vifcor function (Naimi et al. 2014). To test whether the current Bioclim data reflects longer‐term conditions, we also obtained historical bioclimatic data from CHELSA‐TraCE21k (Karger et al. 2023a, 2023b) for the last 2000 years for BIO1, BIO7 and BIO12 (see Figure S1 plotted over time), which was highly correlated with the current 1970–2000 Bioclim data (see Table S4). We then modelled gene expression of all individuals as the response, with the Bioclim variables as the fixed effects and provenance as the random effect using a linear mixed effects model (lmer(gene expression ~ Bioclim variables + (1|Provenance))) for both species. We repeated this model using three datasets: the current Bioclim data (1970–2000), the averaged historical bioclimatic data of the last 2000 years, and the period between 1500 and 1599, which showed abnormalities in temperature changes compared to other periods.

We obtained *C. fumiferana* defoliation data from Gray and MacKinnon (2006) for five of the *P. glauca* provenances in the eastern part of its range. These *C. fumiferana* data were classified as a factorial variable according to Gray and MacKinnon (2006), based on their average cumulated outbreak data between 1941 and 1998, as: 1: very mild, 2: mild, 3: mild–moderate (Provenances 1333 and 1334), 4: moderate (Provenances 1320 and 1329), 5: moderate–severe, 6: severe (Provenance 1325) and 7: very severe, and mapped onto a 2 × 2‐km resolution grid of Canada where provincial forest health survey data was available. As limited provenance‐level information on *C. fumiferana* outbreaks was avaliable, we could not include biotic and abiotic explanatory variables in a single model, as this would overload the model. Therefore, we modelled the effect of outbreak intensity alone on *βglu‐1* gene expression: lm(gene expression ~ outbreak class * provenance), but we further investigated the correlation between *βglu‐1* gene expression, any significant climate variables from the climate analysis, and *C. fumiferana* outbreak class using Spearman correlations.

## 3 Results

### 3.1 *β* *glu‐1* Transcript Level Detection Using RT‐qPCR on Preliminary Sample Set

All three primer pairs for β*glu‐1* were amplified in RT‐qPCR reactions across all populations in both species of the preliminary sample set. In *P. glauca* , we observed significant differences in gene expression between provenances (Table 1) with no provenance by primer pair interactions, indicating that the transcript levels detected were comparable across provenances. Transcript levels for primer pairs *βglu‐1*‐E1 and *βglu‐1*‐E3 were significantly different from each other in both species (Figure S2). This primer effect is likely because the *βglu‐1*‐E3 primer pair amplifies an invariant region (fifth exon–exon junction of the *βglu‐1* gene), whereas the primer pairs *βglu‐1*‐E1 and *βglu‐1*‐E2 both amplify a variable region because of exon 10 being absent in some *βglu‐1* gene forms in both *P. abies* (Piabi_cg1_00079) and *P. glauca* (Pigla_cg1_00089) (Figure 2). Therefore, the primer pairs *βglu‐1*‐E1 and *βglu‐1*‐E3 were used for comparisons to targeted transcriptome sequencing results.

**TABLE 1:** Multiple measures ANOVA to determine provenance and primer pair effects on *β* *glu‐1* transcript levels using RT‐qPCR for the preliminary sample of *Picea glauca* and *Picea abies* populations.

	Effect	DFn	DFd	*F*	*p*	*p* < 0.05
*Picea glauca*	Provenance	8	16	6.32	8.94E‐04	*
	Primer	2	4	10.41	0.03	*
	Provenance: Primer	16	32	10.05	0.44	
*Picea abies*	Provenance	7	14	1.47	0.26	
	Primer	2	4	149.23	1.75E‐04	*
	Provenance: Primer	14	28	1.94	0.07	

### 3.2 *β* *glu‐1* Transcript Level Detection Using Targeted Transcriptome Sequencing and Comparisons With RT‐qPCR Methods on Preliminary Sample Set

The transcriptome sequencing captured 95 of 161 gene targets in *P. glauca* and 106 of 140 targets in *P. abies* . Two genes were significantly differentially expressed between the provenances using a likelihood ratio test of read counts in *P. glauca* ; namely, one of the full‐length gene forms of *βglu‐1* (Pigla_c1g_00044; adjusted *p* value = 5.67E‐05) and one single‐copy gene (Pigla_c1g_00048; adjusted *p* value = 0.007) (Figure S3). However, only Pigla_c1g_00044 had a log fold change > |1|. There were no differentially expressed *βglu‐1* gene forms in *P. abies.* We also did not find any difference in transcript levels for *Ugt5b* or any of the other single‐copy genes across the different populations (Figure S3) in either species.

**FIGURE 3:**
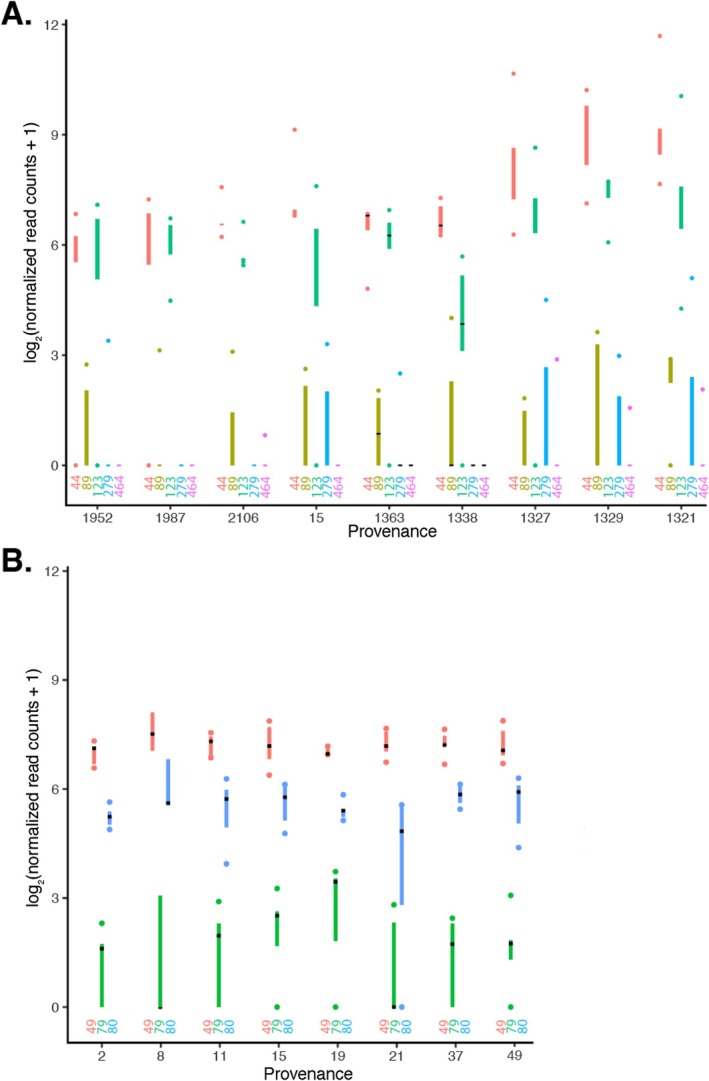
Modified box plot of transcript abundance determined by targeted RNA sequencing for all full‐length and near full‐length *βglu‐1* gene forms for (A) *Picea glauca* with the provenances sorted by longitude (gene forms ‐ 44: Pigla_c1g_00044, 89: Pigla_c1g_00089, 123: Pigla_c1g_00123, 279: Pigla_c1g_00279, and 464: Pigla_c1g_00464), (B) *Picea abies* (gene forms ‐ 49: Piabi_c1g_00049, 79: Piabi_c1g_00079, and 80: Piabi_c1g_00080).

The normalised transcript abundance of *βglu‐1* is presented for all five full‐length and near‐full‐length *βglu‐1* gene forms in *P. glauca* and all three full‐length and near‐full‐length gene forms in *P. abies* (Figure 3). In *P. abies* , one gene form (Piabi_c1g_00049) was consistently more highly expressed across all populations, whereas in *P. glauca* , there was more variability in the expression levels among the gene forms, with Pigla_c1g_00044 and Pigla_c1g_00123 being the most highly expressed. In *P. glauca* , the transcript levels of four of the five gene forms were correlated, and in *P. abies* , two of the three gene forms were correlated (see Table S5).

**FIGURE 4:**
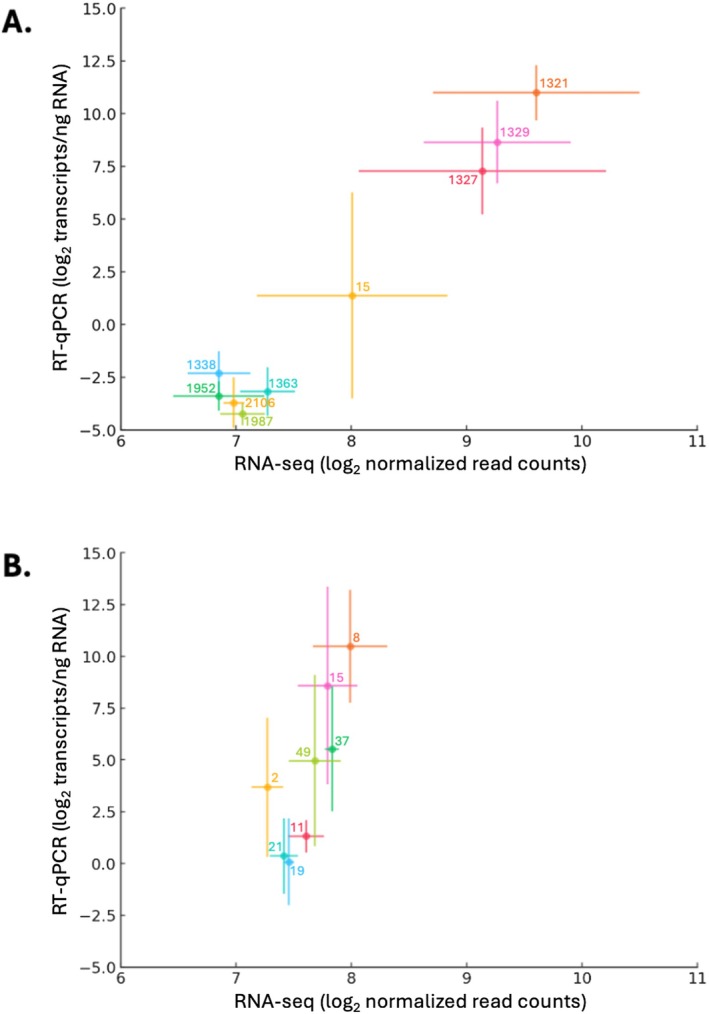
Comparisons of *βglu‐1* transcript levels using targeted RNA sequencing (sum of all gene forms) and RT‐qPCR (average of *βglu‐1‐*E1 and *βglu‐1‐*E3) for the preliminary sample set of (A) *Picea glauca* (nine populations, *n* = 33) and (B) *Picea abies* (eight populations, *n* = 32).

Both RT‐qPCR and targeted transcriptome sequencing methods produced similar trends in *βglu‐1* transcript levels at the population level for *P. glauca* and *P. abies* (Figure 4). In *P. glauca* , Spearman correlations between RT‐qPCR and targeted transcriptome sequencing results showed transcript levels of both primer pairs to be significantly correlated with the read counts of all but one of the full‐length or near‐full‐length *βglu‐1* gene forms and the sum of all gene forms (Table 2). In *P. abies* , however, this correlation is only significant when comparing the transcript levels of the *βglu1‐E3* primer pair to one of the gene forms and the sum of all gene forms.

**TABLE 2:** Spearman correlation between the *β* *glu‐1* transcript levels determined by RT‐qPCR, using primer pairs *β* *glu‐1*‐E1 and *β* *glu‐1*‐E3, and all full‐length and near full‐length gene forms as determined by targeted transcriptome sequencing for *P. glauca* and *P. abies*.

	Primer	Gene form	rho	*p*‐adj	*p* < 0.05
*Picea glauca*	*βglu‐1*‐E1	Pigla_c1g_00044	0.70	1.82E‐05	*
		Pigla_c1g_00089			
		Pigla_c1g_00123	0.68	2.22E‐05	*
		Pigla_c1g_00279	0.54	1.95E‐03	*
		Pigla_c1g_00464	0.44	0.01	*
		Sum of all 5 gene forms	0.79	3.71E‐07	*
	*βglu‐1*‐E3	Pigla_c1g_00044	0.76	8.23E‐07	*
		Pigla_c1g_00089	0.50	4.26E‐03	*
		Pigla_c1g_00123	0.53	3.31E‐03	*
		Pigla_c1g_00279	0.43	0.02	*
		Pigla_c1g_00464	0.27	0.13	
		Sum of all 5 gene forms	0.79	2.92E‐07	*
*Picea abies*	*βglu‐1*‐E1	Piabi_c1g_00049	0.20	0.39	
		Piabi_c1g_00079			
		Piabi_c1g_00080	0.41	0.09	
		Sum of all 3 gene forms	0.31	0.18	
	*βglu‐1*‐E3	Piabi_c1g_00049	0.29	0.16	
		Piabi_c1g_00079	0.09	0.65	
		Piabi_c1g_00080	0.53	0.01	*
		Sum of all 3 gene forms	0.44	0.03	*

*Note:* * Indicates significance at the *p* < 0.05 level.

### 3.3 Landscape‐Level Quantification of *β* *glu‐1* of Full Sample Set

**FIGURE 5:**
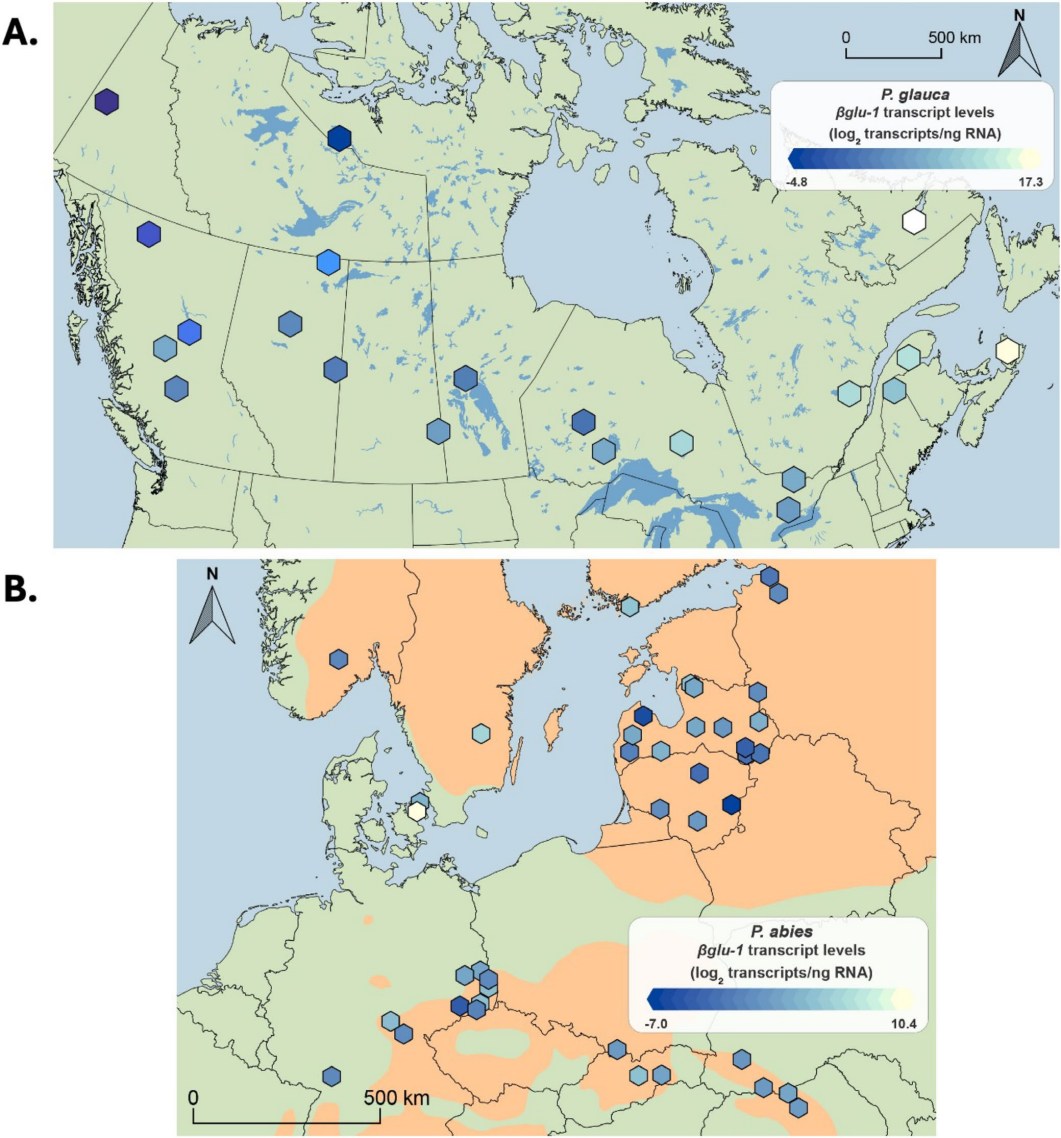
Heatmap showing average gene expression for each provenance of (A) *Picea glauca* (21 populations, *n* = 315; 12 individuals in each provenance), and (B) *Picea abies* (43 populations, *n* = 302) of the full sample set as determined by RT‐qPCR.

For the landscape‐level analysis of RT‐qPCR results, we used the *βglu‐1*‐E3 primer determinations, as it captures the most *βglu‐1* gene forms. We observed substantial within and between provenance variation across the landscape in both species (Figure 5A for *P. glauca* and 5B for *P. abies* ; see Figure S4A for density plot). We observed that *P. glauca* had more variable *βglu‐1* transcript levels, with a very strong longitude effect (Table 3A) and transcript levels increasing eastwards. *P. glauca* also had more individuals with high *βglu‐1* transcript levels compared to *P. abies* , which lacked any geographic pattern. This geographical pattern in *P. glauca* was consistent with the targeted transcriptome sequencing results of the preliminary sample set, whereby eastern *P. glauca* provenances (1321, 1327 and 1329) had the highest transcript levels (cf. Figures 3A, 4A and 5A).

**TABLE 3:** Linear mixed effects model with gene expression as the response, latitude and longitude of the provenances as the fixed effects, and provenance as the random effect variable for (A) *Picea glauca* and (B) *P. abies*.

A.			
Random effects			
Groups		Variance	Std. Dev.
Provenance (Intercept)		10.34	3.22
Residual		23.64	4.86

Fixed Effects					
Variable	Estimate	Std. Error	df	t	*p*
Intercept	36.42	8.04	18	4.53	2.58E‐04*
Latitude	−0.29	0.21	18	−1.38	0.18
Longitude	0.15	0.05	18	3.16	5.40E‐03*

B.		
Random effects		
Groups	Variance	Std. Dev.
Provenance (Intercept)	1.65	1.28
Residual	30.72	5.54

Fixed Effects					
Variable	Estimate	Std. Error	df	*t*	*p*
Intercept	1.84	6.03	30.64	0.31	0.76
Latitude	0.03	0.12	29.97	0.25	0.81
Longitude	−0.12	0.07	34.71	−1.63	0.11

*Note:* * Indicates significance at the *p* < 0.05 level.

Hierarchical clustering of *βglu‐1* transcript levels also showed a separation of individuals from eastern and western provenances (Figure S4B) of *P. glauca* . This observation was further supported by a positive and highly significant (*p* = 2.2E‐6) Moran's I statistic of 0.29, indicating that populations that were geographically close to each other had similar average *βglu‐1* transcript levels. This east versus western provenance clustering was also visualised by NMDS (Figure S5), with a PerMANOVA confirming the clustering to be significant (*p* < 0.001). In contrast, there was no obvious clustering of three genetic clusters described by Chen et al. (2019) of *P. abies* (Figure S4C) and the Moran's I statistic was near zero (0.009) and not quite significant (*p* = 0.06).

### 3.4 Biotic and Abiotic Drivers of Gene Expression

**FIGURE 6:**
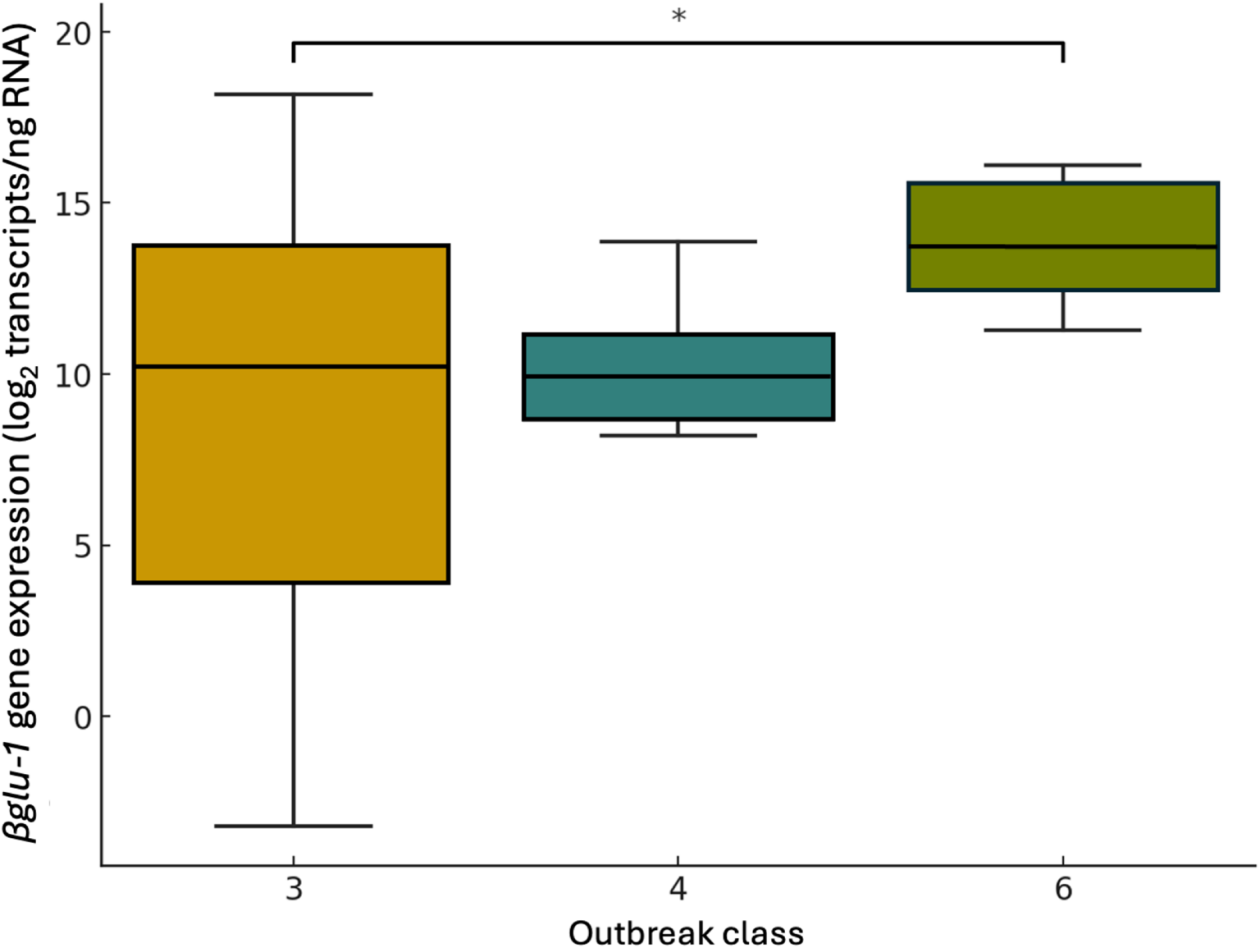
*βglu‐1* transcript levels of five eastern *Picea glauca* provenances across different *Choristoneura fumiferana* outbreak classes according to Gray and MacKinnon (2006). Transcript levels were determined by RT‐qPCR using the *βglu‐1‐E3* primer pair. * Indicates that outbreak class 3 is significantly different from outbreak class 6 (*p* = 5.10E‐03).

Different bioclimatic variables were significantly associated with *βglu‐1* transcript levels in the two species (Table 4). We found mean annual precipitation (BIO12) in *P. glauca* (Table 4A) and temperature annual range (BIO7) in *P. abies* (Table 4B) as significant bioclimatic variables in explaining *βglu‐1* transcript levels. The same variables were identified when modelled using the current (1970–2000) and historical bioclimatic data in both species (see Table S6). Across the *P. glauca* distribution, BIO12 covaries with longitude, with eastern provenances having higher mean annual precipitation than western populations. Therefore, when we modelled the bioclimatic variables for the eastern and western provenances separately, none of the bioclimatic variables remained significant for *P. glauca* (see Table S7). However, we were able to test the effect of outbreak class within the eastern range of *P. glauca* , which showed that provenances that experienced the highest *C. fumiferana* outbreak class (6) had significantly higher *βglu‐1* transcript levels (t = 2.92, *p* = 5.10E‐03; Figure 6). We further verified that *βglu‐1* transcript levels are indeed highly positively correlated with *C. fumiferana* outbreak class (*r* = 0.95, *p* = 0.01), but also found that BIO12 was highly positively correlated with *C. fumiferana* outbreak class (*r* = 0.95, *p* = 0.01), although *βglu‐1* transcript levels were not as significantly correlated with BIO12 (r = 0.80, *p* = 0.10) in these five eastern populations. Therefore, outbreak class may be a more plausible explanatory factor for gene expression patterns in *P. glauca*, indicating that high herbivory pressure could be a selective force on this trait.

**TABLE 4:** Linear mixed effects model with gene expression as the response, Bioclim variables as the fixed effects, and provenance as the random effect variable for (A) *Picea glauca* and (B) *P. abies* .

A.		
Random effects		
Groups	Variance	Std. Dev.
Provenance (Intercept)	12.34	3.51
Residual	23.64	4.86

Fixed Effects					
Variable	Estimate	Std. Error	df	*t*	*p*
Intercept	−6.85	10.18	16	−0.673	0.51
BIO1: Mean annual temperature	0.09	0.33	16	0.278	0.78
BIO7: Temperature annual range	0.01	0.19	16	0.07	0.94
BIO12: Mean annual precipitation	0.02	4.34E‐03	16	3.67	2.06E‐03*
BIO15: Precipitation seasonality	8.68E‐03	0.07	16	0.12	0.91

B		
Random effects		
Groups	Variance	Std. Dev.
Provenance (Intercept)	1.29	1.16
Residual	30.58	5.53

Fixed Effects					
Variable	Estimate	Std. Error	df	*t*	*p*
Intercept	25.46	10.32	32	2.47	0.02*
BIO1: Mean annual temperature	−0.48	0.35	34.35	−1.36	0.18
BIO7: Temperature annual range	−0.70	0.25	29.67	−2.77	9.48E‐03*
BIO12: Mean annual precipitation	−5.52E‐03	3.34E‐03	30.91	−1.65	0.11
BIO15: Precipitation seasonality	0.07	0.06	35.29	1.05	0.30

*Note:* * Indicates significance at the *p* < 0.05 level.

## 4 Discussion

We observed variation in constitutive gene expression associated with acetophenone defence in both *P. glauca* and *P. abies* , as determined through targeted transcriptome sequencing and RT‐qPCR. Our findings were consistent across 2 years of sampling and with RNA‐Seq results on the preliminary sample set. We demonstrate that while both species have within and between population variation in *βglu‐1* transcript levels, a clear geographical pattern was observed only in *P. glauca* , where *βglu‐1* transcript levels increase eastward throughout its range. Abiotic variables explain some variation in gene expression in both species, and in *P. glauca* , there is additional evidence to suggest that a combination of climate and selective pressure from the defoliator, *C. fumiferana* , is important in explaining *βglu‐1* transcript levels.

### 4.1 Variation in *β* *glu‐1* Transcript Levels

Our results clarify the genomic basis of *βglu‐1* transcript levels, supported by complementary data from both RT‐qPCR, which amplifies all *βglu‐1* gene forms, and targeted transcriptome sequencing, which can capture each of the recently described full‐length and near full‐length *βglu‐1* gene forms individually (Hung et al. 2024). We used *βglu‐1* transcript levels as a proxy for the defence phenotype based on the correlation between these levels and acetophenone concentrations reported in Parent et al. (2020). Elevated *βglu‐1* transcript levels suggest a more robust constitutive defence against insect herbivores (Mageroy et al. 2015). Among the genes assessed in *P. glauca* , we observed that the most differentially expressed gene across all populations is one of the full‐length *βglu‐1* gene forms (Pigla_c1g_00044). This suggests that *βglu‐1*, rather than *UGT5b*, which functions upstream of *βglu‐1* and is not differentially expressed, is likely more directly responsible for variation in acetophenone accumulation at the population level.

Moreover, our results show a large variation in constitutive *βglu‐1* expression levels in *P. glauca* and, to a lesser extent, *P. abies* , both within and between populations. Notably, the range of *βglu‐1* transcript levels observed in *P. glauca* aligns with the data reported by Mageroy et al. (2015). However, the large variation observed within populations in both species may suggest that the spatial and temporal variation of selective pressure from *C. fumiferana* leads to fluctuating selection, rather than the fixation of genetic variation (Johnson et al. 2023; Otto and Whitlock 1997). Additionally, there could be an unknown evolutionary trade‐offs associated with this trait (Agrawal 2011; Agrawal and Hastings 2019; see also de Vries et al. (2019) for trade‐offs between insect defence and light competition). Other *Picea* species also have resistant and susceptible phenotypes against other insects (Whitehill et al. 2021), with resistant phenotypes showing higher levels of defence‐related transcripts, while susceptible phenotypes exhibit greater levels of growth‐related transcripts.

### 4.2 Causes of Variation in *β* *glu‐1* Gene Expression at the Molecular Level

Variation in *βglu‐1* expression is likely complex because of its multigenic control (Méndez‐Espinoza et al. 2018; Lamara et al. 2018) and the presence of multiple *βglu‐1* gene forms with high genomic copy number variation. Hung et al. (2024) quantified the genomic copy numbers of the different *βglu‐1* gene forms using the same preliminary sample set. While the gene form with the highest expression in both species corresponds to the highest genomic copy number, no other clear relationship was observed between transcript levels and genomic copy numbers. Our targeted transcriptome sequencing results show differences in RNA transcript levels among the different *βglu‐1* gene forms; however, the relative expression levels of each gene form remain consistent across populations. The complete and full‐length gene forms of *βglu‐1* are the most highly expressed in each species (Pigla_c1g_00044 and Pigla_c1g_00123 for *P. glauca* , and Piabi_c1g_00049 and Piabi_c1g_00080 for *P. abies* ) across all populations. These four gene forms share high sequence similarity and are closely related to the ancestral gene form of *βglu‐1* (Pigla_c1g_00464), which possesses a truncated fourth exon (Hung et al. 2024). Their similar expression levels may be attributed to co‐localisation on the same linkage group, a phenomenon also reported in other systems (reviewed by Michalak 2008).

Sequence variations affecting *βglu‐1* expression may also exist between the distinctive eastern and western haplotypes of *P. glauca* (de Lafontaine et al. 2010) and between the three distinct genetic clusters of *P. abies* (Chen et al. 2019). For example, there may be allelic variation in the promoter region (Huang et al. 2024) or transcription factors that could influence transcription or steady‐state RNA levels in eastern and western populations of *P. glauca* . However, the presence of multiple copies of *βglu‐1* and allelic variation complicates data interpretation, and the transcription factors for *βglu‐1* are currently unknown.

### 4.3 Biotic Selective Forces Explaining Patterns of *β* *glu‐1* Transcript Levels

**FIGURE 7:**
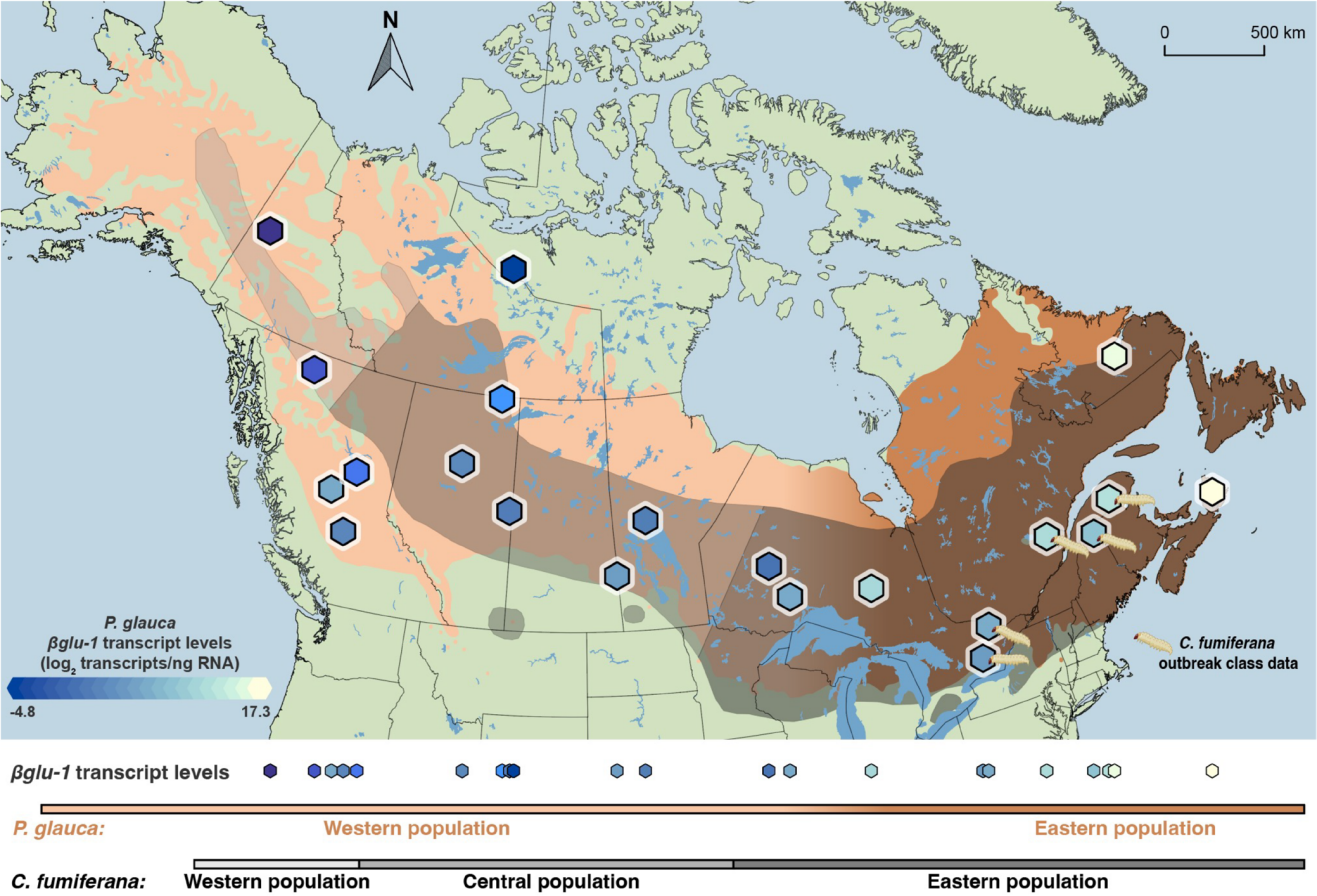
Distribution of *Picea glauca* and *Choristoneura fumiferana* , and the heatmap of *βglu‐1* transcript levels (hexagons). *P. glauca* haplotypes are shaded in beige (western populations) and transitions into brown (eastern populations) based on de Lafontaine et al. (2010). *C. fumiferana* distribution is shaded on a grey scale with different shades representing western (light grey), central (mid‐grey), and eastern (dark grey) populations according to Lumley et al. (2020). *P. glauca* provenances with *C. fumiferama* outbreak data are marked with budworm symbol.

The selective pressure from eastern *C. fumiferana* populations may explain why eastern *P. glauca* populations have maintained high levels of *βglu‐1* expression, as shown in Figure 7. The present population structure of *C. fumiferana* and *P. glauca* in central and eastern North America likely developed during the Holocene (Lumley et al. 2020). During glaciation, both *C. fumiferana* and *P. glauca* persisted in a refugium as single populations, which later diverged during their post‐glacial migration north of the Great Lakes, after the retreat of the Laurentian ice sheets and drainage of Lake Agassiz (Lumley et al. 2020). Likewise, *Picea glauca* populations established their current ranges approximately 12,000–7000 years ago (Payette 1993; de Lafontaine et al. 2010; Anderson et al. 2011), or approximately 140–240 generations (Hornoy et al. 2015) using a conservative generation time estimate of about 50 years (Bouillé and Bousquet 2005). During this period, significant spatial and temporal shifts in the selective pressures from insect pests across the landscape may have already occurred, potentially leading to *Picea* populations gaining or losing the ability to mount a chemical response to defoliators over time.

Although we were not able to obtain *C. fumiferana* outbreak data prior to 1945, *C. fumiferana* outbreaks have been frequent in eastern Canada throughout the Holocene (Morin et al. 2021). Navarro et al. (2018) identified 87 peaks in the abundance of *C. fumiferana* wing scales from lake sediments in a boreal lake in Quebec over the last 10,000 years. Furthermore, frequent and synchronised outbreaks have been documented from historical reconstructions of *C. fumiferana* outbreak history in Quebec and New Brunswick (Royama 1984). The reconstructions date back to at least 700 years in southern Quebec and are based on regional tree ring chronology, where nine potential outbreaks and three uncertain conditional outbreaks were identified since 1564 (Boulanger et al. 2012). However, *C. fumiferana* outbreaks have become more frequent in the 20th century compared to the previous 300 years in eastern Canada, with increasing outbreak frequencies and sizes as they spread northward (Candau and Fleming 2011; Cooke 2014). Recent outbreaks have affected up to 55 million hectares, in contrast to earlier, more localised outbreaks (Blais 1987). This may be why *C. fumiferana* herbivory represents a selective force on acetophenone defence and *βglu‐1* expression levels.

*P. abies* populations are also exposed to various defoliators across their natural range, but none are as prolific as *C. fumiferana* in eastern Canada. Some *P. abies* provenances with high *βglu‐1* transcript levels appear to align with spruce sawfly outbreak areas, *Cephalcia arvensis*, as outlined by Battisti et al. (2000). However, the acetophenone defence in *P. abies* may be less obviously related to geography because of other reasons. The present‐day population structure of *P. abies* has been shaped by human‐mediated transfer of genetic material for forestry purposes, with very little primeval forest remaining (Chen et al. 2019). Therefore, the geographical location of the provenance generally cannot be used as a proxy for the evolutionary origin of *Picea abies* or to determine which of the three genetic clusters it belongs to in most of Northern and Central Europe. This, combined with ongoing gene flow with closely related species (Zhou et al. 2024), could have led to losses of previous local adaptations such as insect resistance.

### 4.4 Abiotic Selective Forces Explaining Patterns of *β* *glu‐1* Transcript Levels

Climate may also be an important explanatory factor to *βglu‐1* transcript levels in both species. In *P. abies* , we identified BIO7 as a possible explanatory variable for *βglu‐1* transcript level, which is consistent with acetophenone concentrations being indicators of plant stress and highly provenance dependent. Lokke (1990) identified drought and nutrient availability as minor single‐impact variables, but hypothesised temperature to be an important explanatory factor. However, no experimental work has been conducted on the effect of temperature on acetophenone defence to date. In *P. glauca* , we find BIO12 to be a significant explanatory variable at the species range level, but not within eastern or western populations, as it correlates with longitude. In addition to BIO12, other unmeasured abiotic variables may also vary longitudinally; therefore, we must remain cautious as to its influence. We also find BIO12 to be positively correlated with *C. fumiferana* outbreak class. It is therefore possible that the influence from *C. fumiferana* outbreak class on *βglu‐1* transcript level is more direct, whereas BIO12 may be more indirect. Future studies should experimentally test the causal relationship between *C. fumiferana* herbivory and *P. glauca* defence phenotype to tease apart these relationships.

Climatic variables that affect plant phenology can also influence herbivory infestation levels (see Mooney et al. 2023; Ekholm et al. 2020). *βglu‐1* transcript accumulation is coupled with the temperature‐dependent budburst timing in *P. glauca* , which in turn influences larval emergence and development timings in *C. fumiferana* (Parent et al. 2017; Pureswaran et al. 2019). Therefore, it is likely that climatic changes such as warmer springs will cause a temporal shift in *βglu‐1* transcript levels in line with variations in the timing of budburst. Budburst may also advance in response to herbivory, as shown in eastern Canadian *P. glauca* (Deslauriers et al. 2019), which has the potential to offset herbivory damage by completing more growth prior to *C. fumiferana* emergence.

### 4.5 Further Perspectives on *β* *glu‐1* Insect Defence

A deeper understanding of the evolution and adaptive variation of acetophenone defences in *Picea* species can inform management practices and provide a long‐term solution to insect herbivory under the changing climate. Generally, the geographic pattern of *βglu‐1* transcript level in *P. glauca* corresponds well with current defoliation patterns of *C. fumiferana* , which likely impose temporally variable selective pressures on the tree host (Berguet et al. 2021), thus resulting in a wide range of transcriptomic responses within and between provenances. For *P. abies* , however, we need to better understand the causes of variation in *βglu‐1* expression, with temperature being a potential explanatory factor, as it lacks a clear geographical structure. This may be in part because we only obtained *P. abies* samples from a reduced part of its range, with samples lacking from the Siberian and north‐eastern parts of its distribution, whereas *P. glauca* sampling is more complete. To date, only a handful of studies have investigated species interactions on gene expression at an ecological scale (see Swenson and Jones 2017). Our findings provide valuable insights into the adaptability of insect defence traits and serve as a baseline for future studies into plant‐insect pest interactions.

## Author Contributions

E.T.Y.W. and J.J.M. designed the study; E.T.Y.W., T.H.H., J.J.M. and A.J. collected the *P.abies* foliage samples; A.U. and N.E. collected the *P.glauca* foliage samples; E.T.Y.W. conducted the laboratory work and T.H.H. conducted the target transcriptome sequencing. E.T.Y.W., H.A. and T.H.H. analysed the data. E.T.Y.W. wrote the manuscript with editorial input from J.J.M., N.E. and S.M.C. All authors have read and given their approval for publication.

## Conflicts of Interest

The authors declare no conflicts of interest.

## Supporting information


**Table S1:** Provenances used in this study for (A) *P. glauca* and (B) *P. abies* , including the number of trees sampled in the preliminary (2021) and full (2022) sample set, as well as the latitude (Lat.) and longitude (Long.) of each provenance.
**Table S2:** Primers for the target gene, *βglu‐1*, and reference gene, *Ef1a*, used for RT‐qPCR. Each primer pair was verified using Sanger sequencing and the amplified region was aligned to the gene to determine if the expected region was amplified. The sequences and the positions are based on Genbank accession # KJ780719 for β*glu‐1* and BT112014 for *Ef1a*.
**Table S3:**—Sequences for synthetic gene fragments synthesised by Twist Biosciences for RT‐qPCR standard curve development. The sequences are based on Genbank accession # KJ780719 for *βglu‐1* and BT112014 for *Ef1a*.
**Figure S1:**. Palaeoclimatic data in 100‐year intervals from 0 (2000 year bp) to 20 (preset day) of Bio 1 (mean annual temperature) for (A) *P. glauca* and (B) *P. abies* , Bio 7 (annual temperature range) for (C) *P. glauca* and (D) *P. abies* , and Bio 12 (mean annual precipitation) for (E) *P. glauca* and (F) *P. abies* .
**Table S4:**. Pearson's correlation between palaeoclimate (2000 y average) and current Bioclim (1970–2000, 30 s) data for all *P. abies* and *P. glauca* provenances.
**Figure S2:**. *βglu‐1* transcript levels of 2021 white and Norway spruce samples as determined by RT‐qPCR using three different primer pairs (from Mageroy et al. 2015) for *P. abies* and *P. glauca* . * Indicate significant pairwise comparisons.
**Figure S3:**. Volcano plot showing the differential gene expression of all genes from target transcriptome sequencing using the likelihood ratio test for *P. glauca* .
**Table S5:** *p* values from Pearson correlations for transcript abundance between the full‐length *βglu‐1* gene forms for (A) *P. glauca* and (B) *P. abies* .
**Figure S4:**. (A) Density plot of the full *P. glauca* and *P. abies* sample set transcript levels across all populations. *βglu‐1* transcript levels were further plotted on a dendrogram using hierarchical clustering for (B) *P. glauca* (eastern provenances = warm colours, western provenances = cool colours) and (C) *P. abies* (northern European provenances = warm colours, southern European provenances = cool colours).
**Figure S5:**. Non‐numeric multi‐dimensional scaling (NMDS) of *P. glauca* provenances, showing clear clustering of *βglu‐1* transcript levels between eastern (white) and western (green) provenances.
**Table S6:** Palaeoclimatic data (past 200 years) and data between the period 1400–1500 for Bioclim variables 1, 7 and 12 modelled with gene expression for *P. glauca* and *P. abies* provenances using a linear mixed effects model. Only *p* values are presented, * indicates significance at *p* < 0.05.
**Table S7:** Current Bioclim data (1970–2000) modelled for western and eastern *P. glauca* provenances separately using a linear mixed effects model. Only *p* values are presented, * indicates significance at *p* < 0.05.

## Data Availability

Sequence data of transcriptomic sequence capture can be publicly accessed in NCBI GenBank under the BioProject PRJNA1172330 (http://www.ncbi.nlm.nih.gov/bioproject/1172330). All other data including normalized read counts, RT‐qPCR data, and Bioclim and insect outbreak data and code can be accessed on Dryad Digital Repository (https://doi.org/10.5061/dryad.k3j9kd5j7).
