# Supplementary material for: Variation in the Molecular Phenotype of βglu‐1, an Insect Defence‐Related Beta‐Glucosidase Gene, in Two Transcontinental Picea Species
Source: Mol Ecol. 2025 Oct 23;34(22):e70120. doi: 10.1111/mec.70120 (PMC12617029; doi:10.1111/mec.70120)
Supplement: Supplementary file 1 — Table S1: Provenances used in this study for (A) P. glauca and (B) P. abies , including the number of trees sampled in the preliminary (2021) and full (2022) sample set, as well as the latitude (Lat.) and longitude (Long.) of each provenance. Table S2: Primers for the target gene, βglu‐1, and reference gene, Ef1a, used for RT‐qPCR. Each primer pair was verified using Sanger sequencing and the amplified region was aligned to the gene to determine if the expected region was amplified. The sequences and the positions are based on Genbank accession # KJ780719 for βglu‐1 and BT112014 for Ef1a. Table S3:—Sequences for synthetic gene fragments synthesised by Twist Biosciences for RT‐qPCR standard curve development. The sequences are based on Genbank accession # KJ780719 for βglu‐1 and BT112014 for Ef1a. Figure S1:. Palaeoclimatic data in 100‐year intervals from 0 (2000 year bp) to 20 (preset day) of Bio 1 (mean annual temperature) for (A) P. glauca and (B) P. abies , Bio 7 (annual temperature range) for (C) P. glauca and (D) P. abies , and Bio 12 (mean annual precipitation) for (E) P. glauca and (F) P. abies . Table S4:. Pearson's correlation between palaeoclimate (2000 y average) and current Bioclim (1970–2000, 30 s) data for all P. abies and P. glauca provenances. Figure S2:. βglu‐1 transcript levels of 2021 white and Norway spruce samples as determined by RT‐qPCR using three different primer pairs (from Mageroy et al. 2015) for P. abies and P. glauca . * Indicate significant pairwise comparisons. Figure S3:. Volcano plot showing the differential gene expression of all genes from target transcriptome sequencing using the likelihood ratio test for P. glauca . Table S5: p values from Pearson correlations for transcript abundance between the full‐length βglu‐1 gene forms for (A) P. glauca and (B) P. abies . Figure S4:. (A) Density plot of the full P. glauca and P. abies sample set transcript levels across all populations. βglu‐1 transcript levels were further plotted on a d [file MEC-34-e70120-s001.docx]

**Supplemental materials:**

**Table S1** – Provenances used in this study for A) *P. glauca* and B) *P. abies*, including the number of trees sampled in the preliminary (2021) and full (2022) sample set as well as the latitude (Lat.) and longitude (Long.) of each provenance.

**A.**

| **Provenance** | **Lat.** | **Long.** | **Year sampled** | **# of trees sampled** |
| --- | --- | --- | --- | --- |
| 10 | 59.8833 | -111.7167 | 2022 | 21 |
| 12 | 54.6333 | -110.2167 | 2022 | 17 |
| 15 | 55.2722 | -114.7801 | 2021, 2022 | 4, 19 |
| 1314 | 53.3333 | -60.4167 | 2022 | 24 |
| 1317 | 46.2667 | -60.6167 | 2022 | 17 |
| 1320 | 47.4500 | -68.2000 | 2022 | 20 |
| 1321 | 47.5386 | -68.2139 | 2021 | 5 |
| 1325 | 48.4167 | -66.2500 | 2022 | 24 |
| 1327 | 48.1679 | -79.4152 | 2021 | 4 |
| 1329 | 48.2833 | -71.0000 | 2021, 2022 | 4, 17 |
| 1333 | 44.4333 | -77.8333 | 2022 | 25 |
| 1334 | 45.6833 | -76.8000 | 2022 | 14 |
| 1337 | 49.6000 | -89.1500 | 2022 | 23 |
| 1338 | 49.0269 | -93.0750 | 2021 | 4 |
| 1349 | 51.2000 | -90.2000 | 2022 | 16 |
| 1350 | 49.3000 | -82.7000 | 2022 | 22 |
| 1355 | 54.1667 | -99.1667 | 2022 | 23 |
| 1363 | 53.9364 | -102.6202 | 2021 | 5 |
| 1365 | 51.6333 | -101.6667 | 2022 | 15 |
| 1369 | 54.4667 | -124.2500 | 2022 | 20 |
| 1370 | 55.2500 | -123.0833 | 2022 | 22 |
| 1373 | 59.1667 | -129.2500 | 2022 | 21 |
| 1376 | 52.4167 | -122.9333 | 2022 | 20 |
| 1380 | 66.0333 | -111.9667 | 2022 | 21 |
| 1386 | 64.0167 | -139.0000 | 2022 | 19 |
| 1952 | 54.3988 | -116.8077 | 2021 | 4 |
| 1987 | 54.1996 | -115.6376 | 2021 | 4 |
| 2106 | 55.3046 | -114.8358 | 2021 | 4 |

**B.**

| **Provenance** | **Lat.** | **Long.** | **Year sampled** | **# of trees sampled** |
| --- | --- | --- | --- | --- |
| A03 | 48.8000 | 23.7500 | 2022 | 10 |
| A04 | 48.0333 | 24.3167 | 2022 | 8 |
| A05 | 54.6333 | 23.9500 | 2022 | 8 |
| A06 | 55.1000 | 22.5167 | 2022 | 8 |
| A07 | 54.8333 | 25.4833 | 2022 | 5 |
| A08 | 55.7500 | 24.4667 | 2022 | 12 |
| A09 | 55.9667 | 26.6167 | 2022 | 7 |
| A11 | 56.6000 | 21.7000 | 2022 | 17 |
| A13 | 56.8500 | 24.7667 | 2022 | 19 |
| A14 | 56.9833 | 21.9667 | 2022 | 11 |
| A16 | 57.8833 | 24.9833 | 2022 | 4 |
| A17 | 59.8500 | 29.8833 | 2022 | 11 |
| A20 | 59.4000 | 30.0333 | 2022 | 11 |
| A24 | 56.6833 | 25.9167 | 2022 | 16 |
| A26 | 56.4667 | 23.0500 | 2022 | 12 |
| B01 | 48.8333 | 19.9333 | 2022 | 3 |
| B02 | 48.7667 | 20.7667 | 2021, 2022 | 5, 11 |
| B03 | 50.3500 | 11.3167 | 2022 | 2 |
| B04 | 49.5333 | 19.2833 | 2022 | 5 |
| B07 | 55.9667 | 12.3500 | 2022 | 7 |
| B08 | 55.7500 | 12.2000 | 2021, 2022 | 3, 3 |
| B11 | 47.9000 | 25.4667 | 2021 | 5 |
| B12 | 51.7833 | 14.4000 | 2022 | 7 |
| B13 | 47.3833 | 25.3833 | 2022 | 6 |
| B15 | 51.7000 | 13.7833 | 2021, 2022 | 5, 3 |
| B16 | 51.4167 | 14.6833 | 2022 | 3 |
| B17 | 50.9667 | 13.5167 | 2022 | 5 |
| B18 | 50.9167 | 14.2167 | 2022 | 5 |
| B19 | 50.8833 | 14.1667 | 2021, 2022 | 5, 9 |
| B20 | 50.6667 | 10.8333 | 2022 | 2 |
| B21 | 49.3333 | 8.5833 | 2021, 2022 | 5, 9 |
| B22 | 51.5667 | 14.7000 | 2022 | 5 |
| B30 | 47.7833 | 25.1333 | 2022 | 8 |
| B32 | 56.6000 | 27.5000 | 2022 | 3 |
| B33 | 57.7833 | 25.1000 | 2022 | 4 |
| B36 | 55.8500 | 27.1833 | 2022 | 7 |
| B37 | 57.0000 | 22.3000 | 2021 | 5 |
| B40 | 57.3833 | 22.6000 | 2022 | 2 |
| B43 | 56.0000 | 26.6000 | 2022 | 3 |
| B46 | 57.2833 | 27.8000 | 2022 | 9 |
| B48 | 60.0333 | 23.0333 | 2022 | 9 |
| B49 | 59.5000 | 8.8000 | 2021, 2022 | 5, 7 |
| B50 | 57.5333 | 15.2667 | 2022 | 5 |

**Table S2** – Primers for the target gene, *βglu-1*, and reference gene, *Ef1a*, used for RT-qPCR. Each primer pair was verified using Sanger sequencing and the amplified region was aligned to the gene to determine if the expected region was amplified. The sequences and the positions are based on Genbank accession # KJ780719 for *βglu-1* and BT112014 for *Ef1a*.

| **Gene** | **Primer** | **Sequence** | **Tm^o^** | **Position of amplified region (bp)** | **Amplification length (bp)** |
| --- | --- | --- | --- | --- | --- |
| *Βglu-1*-E1 | F | GCCCAGGCTAATATATCACAGCAAG | 67.2 | 1143 – 1263 | 120 |
|  | R | GACAGGGTATGGTGATTTCGACAAC | 68.2 |  |  |
|  |  |  |  |  |  |
| *Βglu-1*-E2 | F | TCAACAATCCCACTCCACAAAATTC | 68.5 | 1085 – 1165 | 80 |
|  | R | CCTCTGAAGGTGTTAGCTCTTTTAGGT | 65.6 |  |  |
|  |  |  |  |  |  |
| *Βglu-1*-E3 | F | GTAGTCAAAGAGTCGTGGAGGACATAG | 65.9 | 547 – 643 | 96 |
|  | R | CTACGATCTAGGGATTCACGCAC | 65.7 |  |  |
|  |  |  |  |  |  |
| *Ef1a* | F | GCCCAACACTCTTGGAAGCCCT | 71.4 | 667 – 758 | 91 |
|  | R | CCACCCGACCAACAGGCACA | 73.9 |  |  |

**Table S3** – Sequences for synthetic gene fragments synthesized by Twist Biosciences for RT-qPCR standard curve development. The sequences are based on Genbank accession # KJ780719 for *βglu-1* and BT112014 for *Ef1a*.

| **Gene** | **Length** | **Sequence** |
| --- | --- | --- |
| *Pgβglu-1* | 1521 | ATGGATAGATCTCAAGGTCTATGCTCATCCTGCATATGTTTGGTTTCACTTTATCTTTTCATCGTAGCTGCAGGAACTTCTCCAGATCTCGGTAGAAGCAGCTTCCCAAAGGGTTTCAAATTTGGTGCGGGGTCATCTGCTTATCAGGACGAAGGAGCTGCCCTCGAGGGTGGGAAAGGACCCAGCATTTGGGATACATTCTCCCACATTCAAGGGAAAATTGAAGATGGAAAGAATGGGGATGTTGCGGTAGATCAATACCACCGCTATAAGGAAGATGTTCGACATCTCAAATACATGGGAATGGACCTCTATCGTTTTTCTATTTCCTGGTCGCGCATATTTCCTCAGGGATCACCGAGACACGGGGGAGTCAATGAAGAGGGGATCACTTATTACAATAATCTCATCAACGAGCTGCTCAAGAATGGTATGGAGCCTTTCGTTACACTGTATCACTGGGACATGCCGCAAGCCCTGGAGGACGAGTATGGGGGCTTCCGTAGTCAAAGAGTCGTGGAGGACATAGGCACATTCGCAGAAGAATGTTTTCGAGCTTTCGGAGACCGTGTGAAGTATTGGACAACTATAAACGAACCGCTGACCTTTACATTATATGGCTACGATCTAGGGATTCACGCACCGGGTCGCTGTTCGCCCGGATTTGGGAACTGTACGGCGGGGAATTCTGCAACAGAGCCTTACATTGTAACCCATAATATGCTTCTTGCACATAGCGCCGCTGTCAAAACATATAGGACTAAATACCAGGGGAAGCAGAAAGGTTCCATAGGCATATCACTTGTGGTGAACTGGATGGTGCCATACTCAAACTCTTTGCTTGATCAAGAGGCTACTCAGAGAGCAATCGACTTCAGAATAGGATGGTTTTTGGATCCACTGACGTCGGGGAAGTACCCTGATTCTATGCGAAGCTTAGTGGGAGCACGCTTGCCACGATTTAGCAGCGAAGAGGCCAAAGATCTGAAAGGCTCGTTCGACTTTCTTGGTTATAATTATTATCAAACATACTTTACGGTCAACAATCCCACTCCACAAAATTCCTTGAACACTGACTACGTTCTGGATGCCCAGGCTAATATATCACAGCAAGTAAACGGAGTTTACGTTGGATCCTCTGAAGGTGTTAGCTCTTTTAGGTCGTATCCTGCTGGATTGAGGGAAGTGATAAACTATACCAAGCACCGCTATAGCAATCCTCCCATATATATCACTGAGACAGGGTATGGTGATTTCGACAACGGCACAACCCCGCTTGAAGAAGCATTGCAGGATTCGGGGCGAGTGGAGTACTATTCAAAACATCTATCATCCCTACTTGAAGCCATAAGGGAGGGAGCAGACGTCCGAGGTTATGTGGTTTGGTCGTTTTTGGACGGCTTTGAGTGGGCGAGTGGATATGACTATCGCTTTGGTTTATATTATGTTGATTACAACGACAATTTAAAGCGATATCCCAGGGAATCCGCCAATTGGTTTAGAAACGTCCTCAAGTCATAG |
| *Ef1a* | 330 | GAAGCAAATGATTTGTTGCTGTAACAAGATGGATGCCACTACTCCCAAGTACTCAAAGGCTCGTTACGATGAAATTGTCAAGGAAGTATCTTCTTATTTGAAGAGGGTTGGATACAACCCAGACAAGATTCCTTTTGTGCCTATCTCTGGTTTTGAGGGTGACAACATGATTGAGCGCTCGAACAACCTTGATTGGTACAAGGGCCCAACACTCTTGGAAGCCCTTGACCAGGTTTCGGAGCCTAAGCGTCCCACAGACAGCCTCTGAGGTTGCCCCTTCAGGATGTTTACAAAATTGGTGGTATTGGGACTGTGCCTGTTGGTCGGGT |


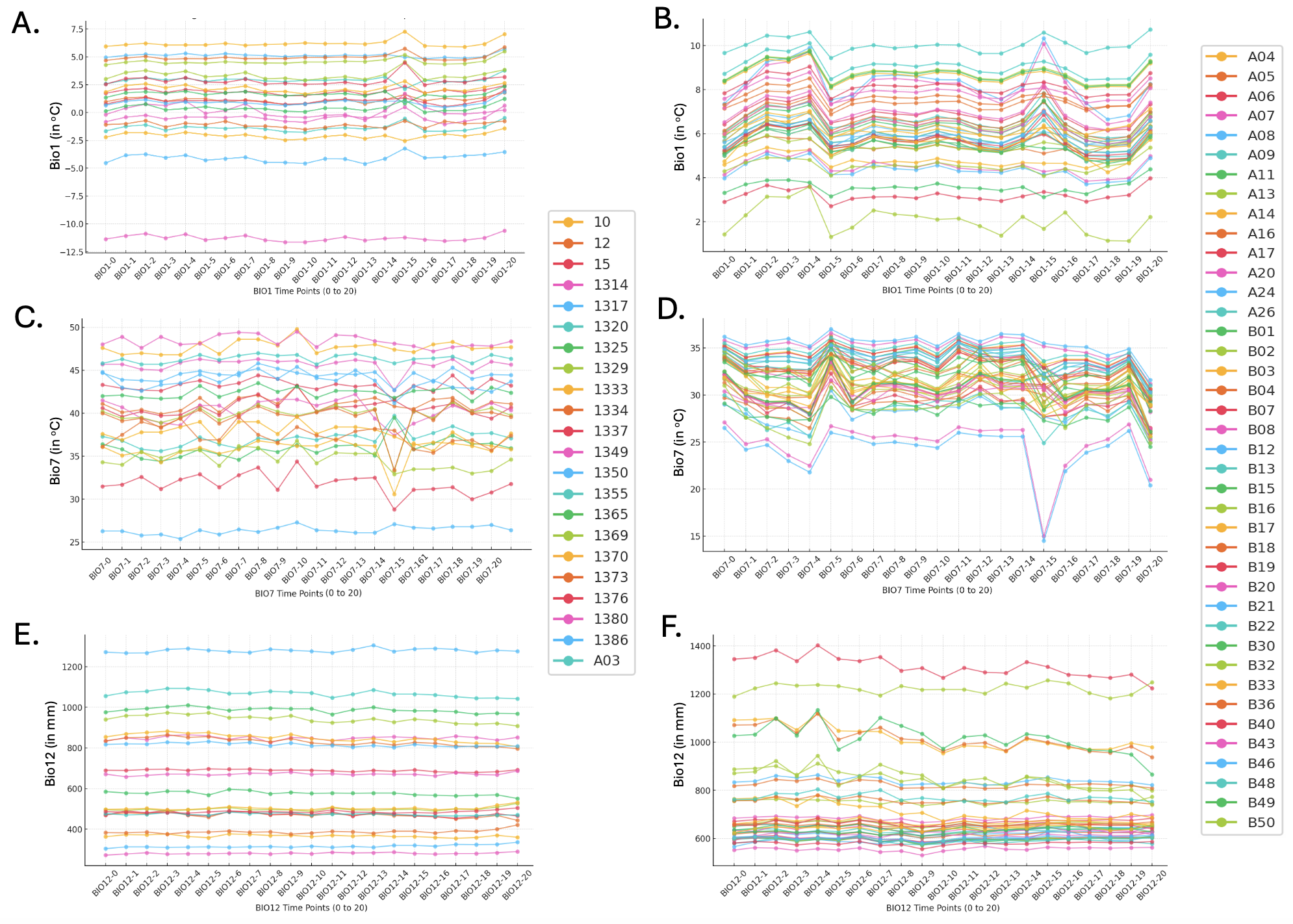


**Figure S1** – Paleoclimatic data in 100-year intervals from 0 (2000 yr bp) to 20 (preset day) of Bio 1 (mean annual temperature) for A) *P. glauca* and B) P*. abies*, Bio 7 (annual temperature range) for C) *P. glauca* and D) P*. abies*, and Bio 12 (mean annual precipitation) for E) *P. glauca* and F) P*. abies*.

**Table S4** - Pearsons correlation between paleoclimate (2000 years average) and current Bioclim (1970-2000, 30 seconds) data for all *P. glauca* and *P. abies* provenances.

|  | **Cor** | **P-value** |
| --- | --- | --- |
| **BIO1** | 0.9938997 | < 2.2e-16 |
| **BIO7** | 0.9328052 | < 2.2e-16 |
| **BIO12** | 0.9360182 | < 2.2e-16 |

**
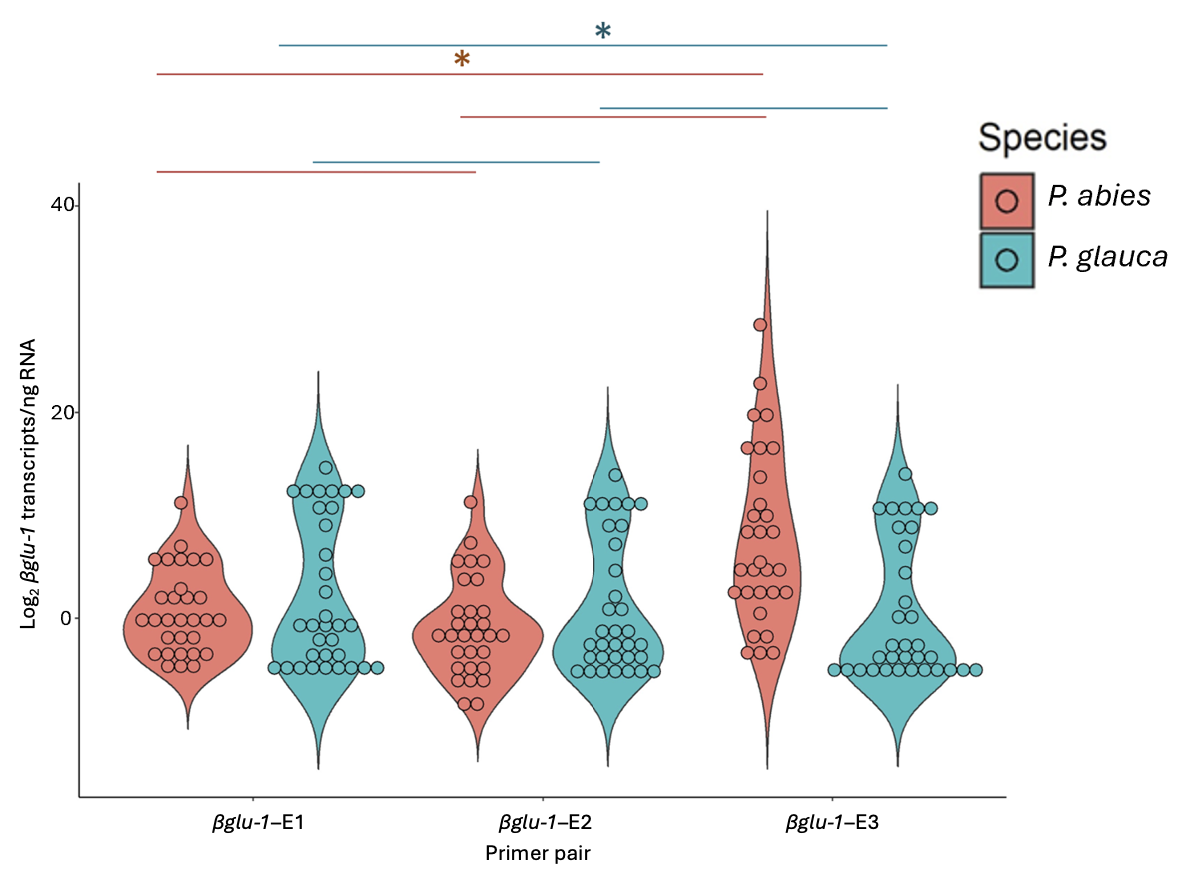
**

**Figure S2** – *βglu-1* transcript levels of 2021 white and Norway spruce samples as determined by RT-qPCR using three different primer pairs (from Mageroy et al. 2014) for *P. abies* and *P. glauca*. * Indicate significant pairwise comparisons.


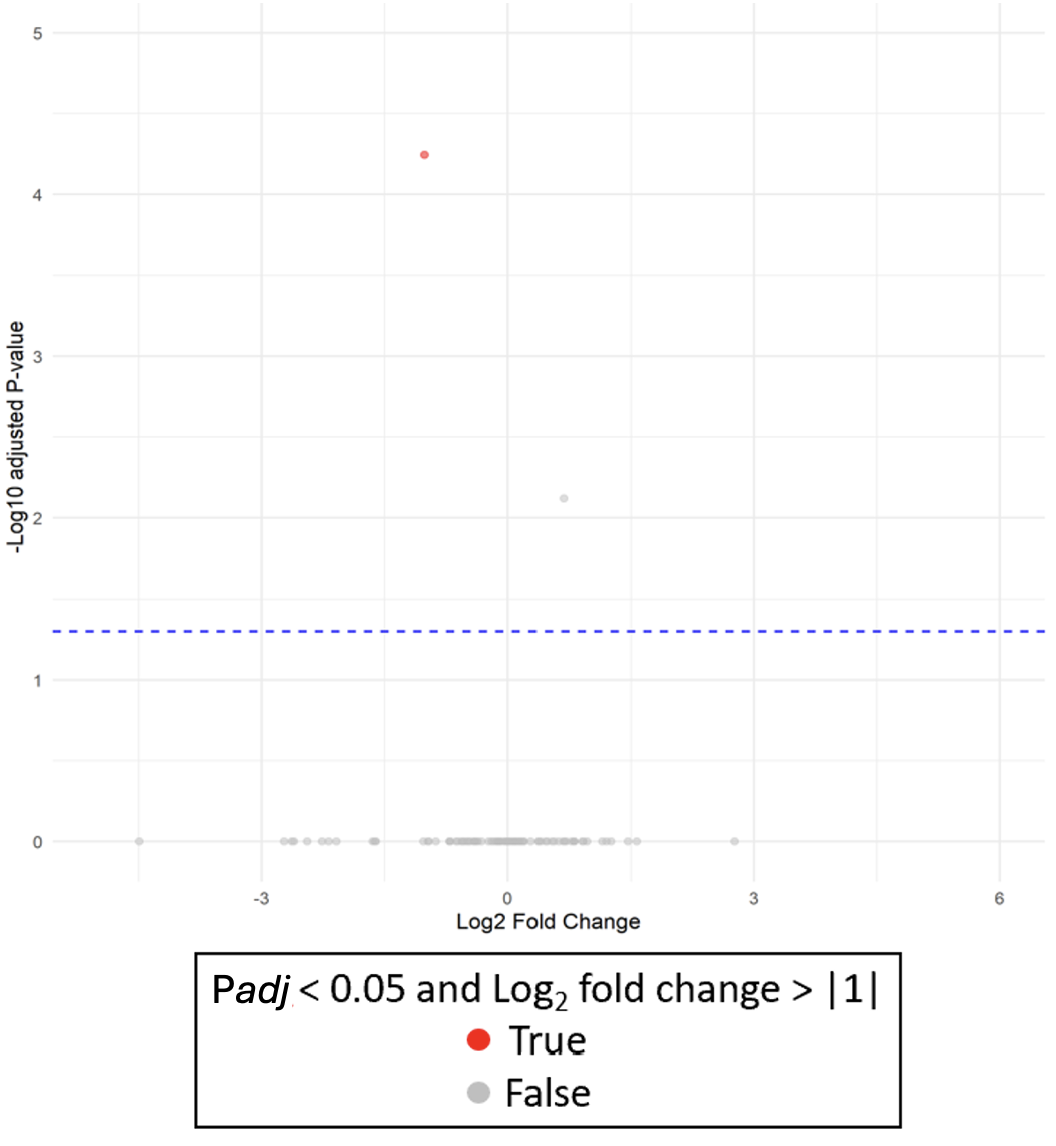


**Figure S3** – Volcano plot of showing the differential gene expression of all genes from target transcriptome sequencing using likelihood ratio test for *P. glauca*.

**Table S5** – P-values from Pearson correlations for transcript abundance between the full length *βglu-1* gene forms for A) *P. glauca* and B) *P. abies*.

| **A.** |  |  |  |  |  |
| --- | --- | --- | --- | --- | --- |
|  | Pigla_c1g_00044 | Pigla_c1g_00089 | Pigla_c1g_00123 | Pigla_c1g_00279 | Pigla_c1g_00464 |
| Pigla_c1g_00044 |  | 0.03 | 2.20E-16 | 2.83E-11 | 2.77E-06 |
| Pigla_c1g_00089 |  |  | 0.12 | 0.36 | 0.4 |
| Pigla_c1g_00123 |  |  |  | 1.67E-13 | 4.96E-05 |
| Pigla_c1g_00279 |  |  |  |  | 1.08E-06 |
| Pigla_c1g_00464 |  |  |  |  |  |
|  |  |  |  |  |  |
| **B.** |  |  |  |  |  |
|  | Piabi_c1g_00049 | Piabi_c1g_00079 | Piabi_c1g_00080 |  |  |
| Piabi_c1g_00049 |  | 0.84 | 2.12E-03 |  |  |
| Piabi_c1g_00079 |  |  | 0.79 |  |  |
| Piabi_c1g_00080 |  |  |  |  |  |


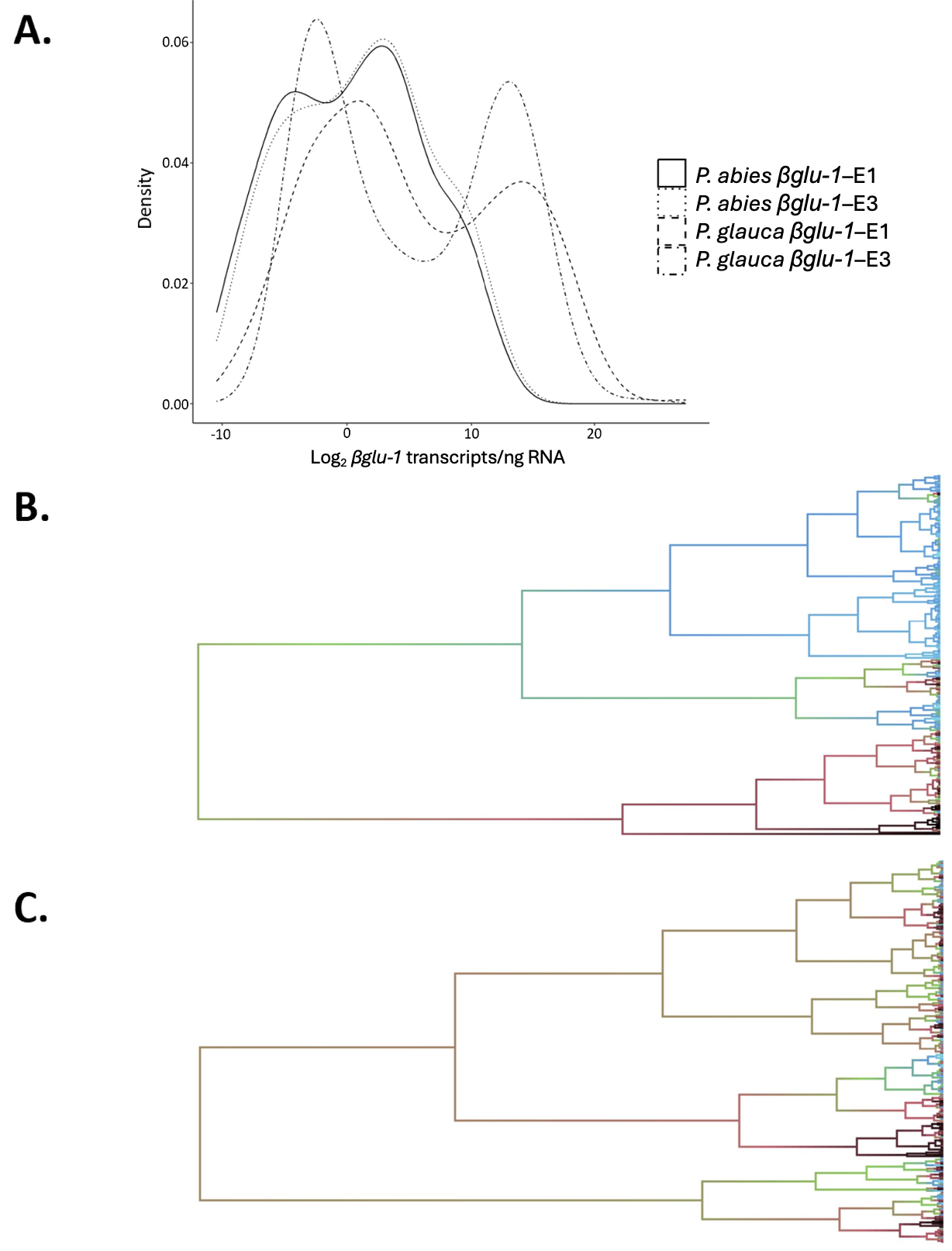


**Figure S4** – A) Density plot of the full *P. glauca* and P. abies sample set transcript levels across all populations. *βglu-1* transcript levels were further plotted on a dendrogram using hierarchical clustering for B) *P. glauca* (eastern provenances = warm colours, western provenances = cool colours) and C) *P. abies* (northern European provenances = warm colours, southern European provenances = cool colours).

**
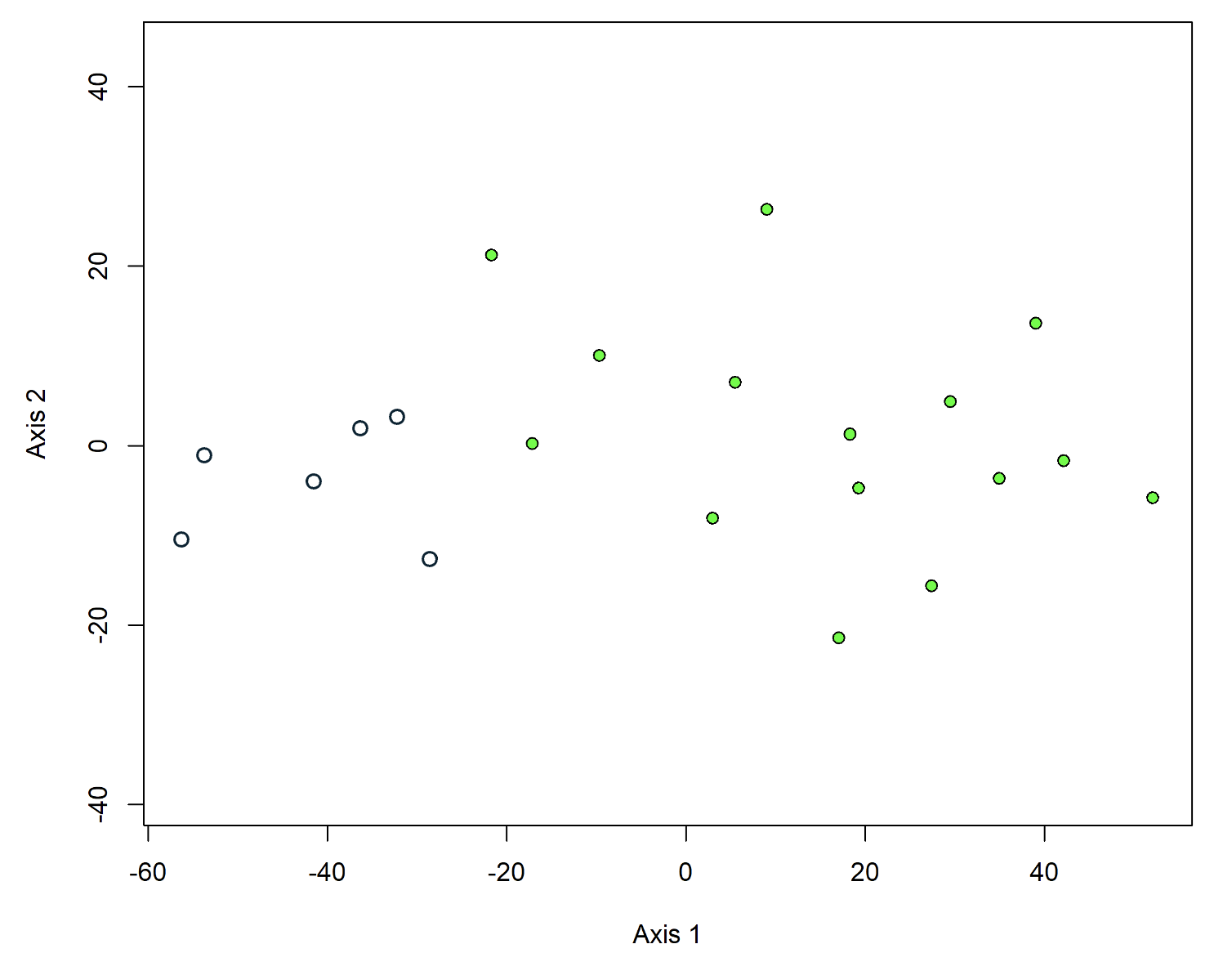
Figure S5** – Non-numeric multi-dimensional scaling (NMDS) of *P. glauca* provenances, showing clear clustering of *βglu-1* transcript levels between eastern (white) and western (green) provenances.

**Table S6** – Paleoclimatic data (past 200 years) and data between the period 1400 - 1500 for Bioclim variables 1, 7, and 12 modelled with *βglu-1* gene expression for *P. glauca* and *P. abies* provenances using a linear mixed effects model. Only p-values are presented, * indicate significance at p < 0.05.

|  | ***P. glauca*** | |  | ***P. abies*** | |
| --- | --- | --- | --- | --- | --- |
|  | **Paleoclimate** | **1500~1599** |  | **Paleoclimate** | **1500~1599** |
| **BIO1** | 0.7 | 0.5 |  | 0.12 | 0.18 |
| **BIO7** | 0.9 | 0.74 |  | 1.53E-03* | 1.45E-03* |
| **BIO12** | 3.46E-05* | 1.42E-05* |  | 0.12 | 0.16 |

**Table S7** – Current Bioclim data (1970 – 2000) modelled for western and eastern *P. glauca* provenances separately using a linear mixed effects model. Only p-values are presented, * indicate significance at p < 0.05.

|  | ***P. glauca*** | |
| --- | --- | --- |
|  | **Western Provenances** | **Eastern Provenances** |
| **BIO1** | 0.11 | 0.11 |
| **BIO7** | 0.51 | 0.65 |
| **BIO12** | 0.99 | 0.16 |
| **BIO15** | 0.97 | 0.17 |
